# Proteomic Analysis and Functional Validation of a *Brassica oleracea* Endochitinase Involved in Resistance to *Xanthomonas campestris*

**DOI:** 10.3389/fpls.2019.00414

**Published:** 2019-04-12

**Authors:** Cristiane Santos, Fábio C. S. Nogueira, Gilberto B. Domont, Wagner Fontes, Guilherme S. Prado, Peyman Habibi, Vanessa O. Santos, Osmundo B. Oliveira-Neto, Maria Fatima Grossi-de-Sá, Jesus V. Jorrín-Novo, Octavio L. Franco, Angela Mehta

**Affiliations:** ^1^Embrapa Recursos Genéticos e Biotecnologia, Brasília, Brazil; ^2^Departamento de Biologia, Universidade Federal de Juiz de Fora, Juiz de Fora, Brazil; ^3^Proteomics Unit, Chemistry Institute, Universidade Federal do Rio de Janeiro, Rio de Janeiro, Brazil; ^4^Departamento de Biologia Celular, Universidade de Brasília, Brasília, Brazil; ^5^Department of Bioprocess Engineering and Biotechnology, Universidade Federal do Paraná, Curitiba, Brazil; ^6^Departamento de Bioquímica e Biologia Molecular, Escola de Medicina, Faculdades Integradas da União Educacional do Planalto Central, Brasília, Brazil; ^7^Centro de Analises Proteomicas e Bioquimica, Pós-Graduação em Ciências Genômicas e Biotecnologia, Universidade Católica de Brasília, Brasília, Brazil; ^8^Department of Biochemistry and Molecular Biology, Universidad de Córdoba, Córdoba, Spain; ^9^S-Inova Biotech, Pós-Graduação em Biotecnologia, Universidade Católica Dom Bosco, Campo Grande, Brazil

**Keywords:** LC-MS/MS, differential protein abundance, qRT-PCR, gene overexpression, plant–pathogen interaction

## Abstract

Black rot is a severe disease caused by the bacterium *Xanthomonas campestris* pv. *campestris* (Xcc), which can lead to substantial losses in cruciferous vegetable production worldwide. Although the use of resistant cultivars is the main strategy to control this disease, there are limited sources of resistance. In this study, we used the LC-MS/MS technique to analyze young cabbage leaves and chloroplast-enriched samples at 24 h after infection by Xcc, using both susceptible (Veloce) and resistant (Astrus) cultivars. A comparison between susceptible Xcc-inoculated plants and the control condition, as well as between resistant Xcc-inoculated plants with the control was performed and more than 300 differentially abundant proteins were identified in each comparison. The chloroplast enriched samples contributed with the identification of 600 additional protein species in the resistant interaction and 900 in the susceptible one, which were not detected in total leaf sample. We further determined the expression levels for 30 genes encoding the identified differential proteins by qRT-PCR. *CHI-B4 like* gene, encoding an endochitinase showing a high increased abundance in resistant Xcc-inoculated leaves, was selected for functional validation by overexpression in *Arabidopsis thaliana.* Compared to the wild type (Col-0), transgenic plants were highly resistant to Xcc indicating that *CHI-B4 like* gene could be an interesting candidate to be used in genetic breeding programs aiming at black rot resistance.

## Introduction

Black rot, caused by the bacterium *X. campestris* pv. *campestris* (Xcc), is one of the most severe diseases that affects cruciferous crops. The use of resistant cultivars is the most efficient strategy to control black rot and therefore, resistance genes have been studied in *Brassica* genomes including the genome A (*B. rapa*), genome BC (*B. carinata*, originated from *B. nigra x B. oleracea*), and genome AC (*B. napus*, originated from *B. rapa* × *B. oleracea*). It is known that the characterized resistance genes can confer durable resistance to black rot in genomes A and B (Guo et al., 1991; Ignatov et al., 2000). However, there is limited information on resistance genes from genome C (*B. oleracea*), and there are no reports on resistance sources against black rot disease for this genome group (Camargo et al., 1995; Saha et al., 2016; Sharma et al., 2016).

Functional genomic and proteomic techniques have been important tools for exploring and understanding plant–pathogen interaction mechanisms. Proteomic studies can provide the link between gene expression and protein abundance and help identify key proteins involved in plant defense and resistance (Kamal et al., 2010a,b; Komatsu and Hossain, 2017). Although mass spectrometer sensitivity and software development have improved protein identification in the last years, there are still some limitations in the detection of low abundant proteins. One alternative to overcome this problem is the analysis of subcellular proteomes. This strategy can reduce sample complexity and provide the identification of a high amount of additional proteins contributing to a better understanding of the metabolic pathways involved (Stekhoven et al., 2014; Wang and Komatsu, 2016). Indeed, analyses of subcellular proteomes have been widely performed and presented a better picture of differential protein abundance under different stress conditions (Peltier et al., 2000; Uberegui et al., 2015). Chloroplasts have an important role in stress response and therefore the study of the chloroplast proteome can bring important contributions for the elucidation of plant defense, especially since this organelle participates actively in plant immune response (Audran et al., 2016).

In a previous study, Ribeiro et al. (2018) analyzed *B. oleracea* leaves inoculated with Xcc by 2-DE. Although differential protein spots were detected, the 2-DE technique is highly limited, particularly in the detection of low abundant proteins (Corthals et al., 2000; Wang and Hanash, 2003). Therefore, in this study, we performed bottom-up proteomics of inoculated leaves at the same time point (24 h after infiltration), in order to understand protein abundance at an early stage of infection. The total leaf proteome was further complemented with the analysis of chloroplast-enriched samples and the expression levels for 30 genes encoding the identified differential proteins were determined by qRT-PCR. Additionally, one protein was selected for overexpression in *Arabidopsis thaliana* to verify its involvement in resistance to Xcc.

## Materials and Methods

### Plant Material and Chloroplast Isolation

In this work, two *B. oleracea* var. *capitata* cultivars, one moderately resistant (Astrus Plus – Chile/Seminis^®^) and one susceptible (Veloce – Brazil/Agristar^®^), as determined previously by our group (data not published), were used. A schematic figure of the experimental design is presented in the Supplementary Figure S1. The isolate of *X. campestris* pv. *campestris* (Xcc) Xcc51, obtained from Embrapa Hortaliças, Brasília, DF, Brazil, was used. Young plants (45 days after sowing) of both cultivars were inoculated with bacterial or saline (0.85% NaCl) solution, according to Santos et al. (2017). Leaves were harvested 24 hours after infiltration (hai), ground in liquid nitrogen and stored at -80°C. Three biological replicates, composed of five plants each, were analyzed. The same samples were used for chloroplast isolation using 5 g of ground material according to Kley et al. (2010), with modification in the percoll gradient. A 40%:80% percoll (Sigma-Aldrich) gradient was used.

### Protein Extraction and LC-MS/MS Analysis

Leaves (0.3 g) and isolated chloroplasts (500 μL) were used for protein extraction according to Mot and Vanderleyden (1989), with modifications, as follows: for chloroplast protein extraction, we used a 1:2 extraction buffer (0.7 M sucrose, 0.5 M Tris-HCl, 50 mM EDTA, 0.1 M KCl, and 40 mM DTT):phenol proportion. Precipitated proteins were solubilized in urea/thiourea buffer (7 M urea; 2 M thiourea; 4% CHAPS, and 5 mM DTT) and quantified using the Bradford Reagent (Bio-Rad, Unite States). Approximately 150 μg of proteins from three biological replicates were loaded onto SDS-PAGE and allowed to migrate approximately 1 cm (Supplementary Figure S2) in a 12% resolving gel, as described by Valledor and Weckwerth (2014). Each gel lane containing one biological replicate was cut and submitted to *in gel* digestion using 5 μg of trypsin (Promega, Madison, WI, Unite States), according to Valledor and Weckwerth (2014). After the digestion procedure, the proteins were quantified using Quibt^TM^ fluorometer (Invitrogen), following the manufacturer’s instructions. Three biological and three technical replicates were analyzed by LC-MS/MS, totalizing nine technical replicates. The peptide samples were desalted according to the protocol described by Rappsilber et al. (2007) and suspended in 50 μL of 4% (v/v) acetonitrile (ACN) and 0.25% (v/v) formic acid.

A total of 2 μg of digested peptides were loaded into a one-dimensional (1D) nano-flow LC-MS/MS system (Thermo Scientific). Peptides were eluted using a monolithic C18 column Acclaim PepMap (Thermo Scientific) of 150 mm in length and 0.075 mm internal diameter. The gradient employed 0.1% formic acid in mobile phase A and 0.1% formic acid and 90% acetonitrile in mobile phase B during 180 min with a controlled flow rate of 400 nL/min from 5 to 35% phase B. The effluent from the nLC column was directly electrosprayed into an Orbitrap Mass Spectrometer (LTQ-Orbitrap XL^TM^ Hybrid Ion Trap, Thermo Scientific), operated in the positive ion mode and set to data-dependent acquisition.

Precursor peptides were detected in the mass range of 400–1,500 m/z and at a resolution of 120 K (at 200 m/z) with a target ion counting of 5 × 10^5^. Tandem MS was performed by the isolation window of 1 atomic mass unit (amu), with CID (collision-induced dissociation) fragmentation in the quadrupole with a normalized collision energy of 35. The automatic gain control (AGC) was defined at 4 × 10^5^ and the max injection time was of 50 ms. Only the 10 most intense precursors in the charge states of 2–6 were subjected to MS2. The dynamic exclusion duration was defined as 15 s with mass error tolerance around 10 ppm. The instrument was operated in max speed mode with cycles of 3 s.

### Protein Identification and Quantification

The raw data were processed using the software Progenesis QI (Nonlinear Dynamics, Waters, Durham, NC, United States) and PEAKS^®^ 7 (Bioinformatics Solutions Inc., Waterloo, ON, Canada). A total of four pairwise comparisons (Xcc-inoculated vs. saline solution-inoculated) were performed (Supplementary Figure S3): (1) resistant Xcc-inoculated leaves compared to saline solution-inoculated leaves (LRI:LRC), (2) resistant Xcc-inoculated chloroplast compared to saline solution-inoculated chloroplast (ChRI:ChRC), (3) susceptible Xcc-inoculated leaves compared to saline solution-inoculated leaves (LSI:LSC), (4) susceptible Xcc-inoculated chloroplast compared to saline solution-inoculated chloroplast (ChSI:ChSC). The chromatograms from each comparison were automatically aligned and the alignment was manually revised for inconsistencies. Profile data from the MS scans were used to calculate the relative peptide abundance using the areas under the peaks of extracted ion chromatograms. Quantified features were median normalized and evaluated for statistical significance using ANOVA *p* ≤ 0.05.

MS/MS files were exported as Mascot generic file (mgf) for peptide identification using PEAKS^®^7 (Bioinformatics Solutions Inc., Waterloo, ON, Canada) software (Zhang et al., 2012), and searched against the UniProt_*Brassica oleracea* database (taxonomy ID 3712) on February 2017^1^. The analysis using PEAKS^®^7 was performed with the following parameters: peptide m/z tolerance – 10 ppm; fragment ion m/z tolerance – 0.5 Da; digestion using trypsin with two missed cleavages allowed; Cys carbamidomethylation as fixed modification and Met oxidation as variable modification. The search results were filtered by FDR < 1%. The SPIDER tool within the software PEAKS^®^7 was used to find homologous peptides presenting a single amino acid substitution in the database (Han et al., 2005). The data generated was deposited in the MassIVE repository (DOI: 10.25345/C5KG6W).

### qRT-PCR Analysis

Total RNA was extracted from the same leaf samples (0.1 g) used for protein analysis by the TRIzol^TM^ Reagent method (Invitrogen^TM^), following the manufacturer’s instructions. RNA samples were quantified using a NanoDrop^TM^ 200 spectrophotometer (Thermo Scientific) and the integrity of the RNA was observed in 1% agarose gel. RNA was treated with Turbo^TM^ DNAse (Applied Biosystems/Ambion) and cDNA synthesis was performed using 2 μg of total RNA and the Go Script^TM^ Reverse Transcription System (Promega), following the manufacturer’s instructions. A total of 30 genes encoding the differentially abundant proteins identified were selected for qRT-PCR (Supplementary Table S1). *SAND* (*SAND family protein*), *TBP1* (*TATA-box-binding protein 1*), *TUB6* (*Tubulin beta-6*), and *UBQ1* (*Ubiquitin-60S ribosomal protein L40*) were used as reference genes. All primers used were designed using Primer3Plus program (Untergasser et al., 2007). qRT-PCR was performed using three biological and three technical replicates, as described by Santos et al. (2017). The analysis was performed in a thermal cycler 7300 Real-Time PCR System (Applied Biosystems). To verify the absence of genomic DNA in the samples, qRT-PCR was performed using RNA as template. For stability evaluation of the reference genes, the geNorm algorithm (Etschmann et al., 2006) was used and the expression analysis was performed with the REST software (Pfaffl et al., 2002).

### Overexpression of *BoCHI-B4 Like* Gene in *Arabidopsis thaliana*

The gene *BoCHI-B4 like* (GAQY01039586) encoding the basic endochitinase CHB4-like protein (A0A0D3BPL2) was selected for functional validation. The binary vector pBin61 that carries a transcription cassette with the *CaMV 35S* promoter and terminator, and the kanamycin resistance gene as selection marker was used (Bendahmane et al., 2002) The *BoCHIB4 like* gene (*BoCHIB4)* was synthesized and cloned into the pBIN61 vector by Epoch Life Science Inc. (Missouri City, TX, United States) to generate the construct pBIN61: *BoCHIB4*, which was used to transform *A. thaliana* (Col-0), mediated by *Agrobacterium tumefaciens* (strain GV3130) using the floral dip method (Bent and Clough, 1998). Approximately 0.5 g of seeds of the transformed plants were sterilized and distributed in MS culture medium supplemented with kanamycin (50 mg L^-1^) resulting in the selection of 15 pBIN61:*BoCHIB4* primary transformants. The parental lines and T2-generation were germinated in MS medium containing kanamycin and transferred to substrate and maintained in a growth chamber under a 12 h light:12 h dark photoperiod at 22°C. To confirm transformation, leaves were harvested for DNA extraction, followed by PCR amplification and sequencing using specific primers. DNA of non-transformed plants (wild type Col-0) was used as control.

### Molecular and Phenotypical Characterization of *Arabidopsis* Transgenic Lines

Leaves of T2 homozygous events were harvested for DNA and RNA extraction for Southern blot and qRT-PCR analysis. For Southern blot, DNA extracted from T2-generation plants (10 μg) was digested with *Xba*I and analyzed using standard procedure (Romano and Vianna, 2015). The RNA preparation and qRT-PCR analysis were performed as described above. The *A. thaliana* reference genes *ACT2* (*Actin 2*) and *EF-1α* (*elongation factor-1α*) were used (Supplementary Table S1). The effect of gene overexpression was confirmed by spraying bacterial solution (Xcc51 OD_600_ = 0.1) followed by disease development scoring from 1 to 5 days post inoculation (dpi) using a disease index ranging from 0 (no symptom, considered highly resistant) to 4 (full leaf necrosis, classified as highly susceptible), based on (Meyer et al., 2005). A total of five events was evaluated and 15 plants from each event were analyzed, as well as 15 wild type Col-0 plants, used as the control.

## Results and Discussion

### Proteomic Profile and Gene Expression Analysis of Brassica Leaves and Chloroplast Enriched Samples

**FIGURE 1:**
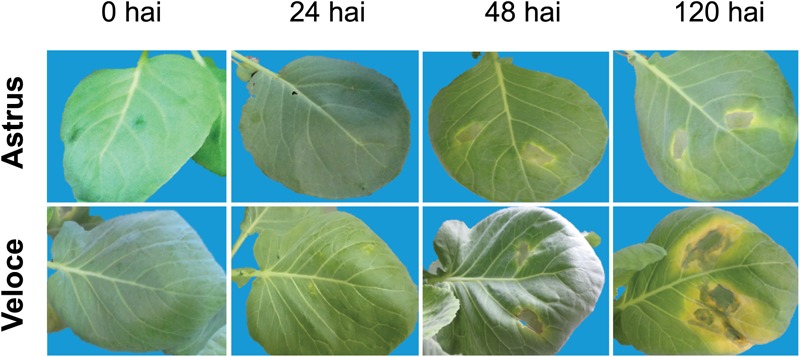
Symptoms of *Brassica oleracea* cultivars Astrus (moderately resistant) and Veloce (highly susceptible), at different hours after inoculation (hai) with *Xanthomonas campestris* pv. *campestris*.

In this study, two conditions were compared for the identification of differentially abundant proteins: Xcc-inoculated and saline solution-inoculated (control condition) leaf and chloroplast from resistant and susceptible cultivars (Astrus and Veloce), resulting in four comparisons (Supplementary Figure S3). As observed in our previous studies (data not published), Astrus was moderately resistant to Xcc51 and Veloce was highly susceptible (Figure 1). The LC-MS/MS data analysis resulted in more than 30,000 peptide sequences, corresponding to more than 1,000 protein species (Supplementary Table S2). Since several of the matches corresponded to uncharacterized proteins in the Uniprot database, a second analysis was performed using Blas2GO software to identify the proteins and infer the gene ontology (GO) for biological process. The leaf proteome of the resistant cultivar inoculated with Xcc (LRI) and in the control condition (LRC) revealed a total of 1,424 proteins, while the leaf samples of the susceptible cultivar Veloce inoculated with Xcc (LSI) and in the control condition (LSC) revealed a total of 1,395 proteins (Supplementary Figure S3).

Chloroplast enriched samples from the resistant cultivar inoculated with Xcc (ChlRI) and in the control condition (ChlRC) as well as from the susceptible cultivar Veloce inoculated with Xcc (ChlSI) and in the control condition (ChlSC) were also analyzed (Supplementary Figure S3). The proteins identified in the leaf and chloroplast enriched samples from each cultivar were merged into a single table (Supplementary Table S2) for discussion, totalizing 2,086 proteins in the resistant interaction, referred to as RI:RC and 2037 in the susceptible interaction, referred to as SI:SC (Supplementary Figure S3). Proteins with the same name were aligned for sequence comparison (protein sequence alignment) using ClustalOmega^2^ and when differences in the sequences were observed they were considered as different protein species. The results showed that the proteome from chloroplast samples contributed with 662 additional proteins in the resistant plants (ChlRI and ChlRC) and 642 in the susceptible (ChlSI and ChlSC) (Supplementary Figure S3), which were not detected in leaf samples. Moreover, a total of 338 differentially abundant proteins were identified in the resistant interaction (RI:RC), 200 of which were obtained from chloroplast enriched samples (Xcc-inoculated compared to the control). The susceptible interaction (SI:SC) revealed 361 differential proteins out of which 175 were identified in chloroplasts (Supplementary Table S3). These results emphasize that the analysis of subproteomes can contribute significantly for the identification of additional proteins (Rolland et al., 2012; Bayer et al., 2015), especially those present in lower abundance (Kim and Kang, 2008).

**FIGURE 2:**
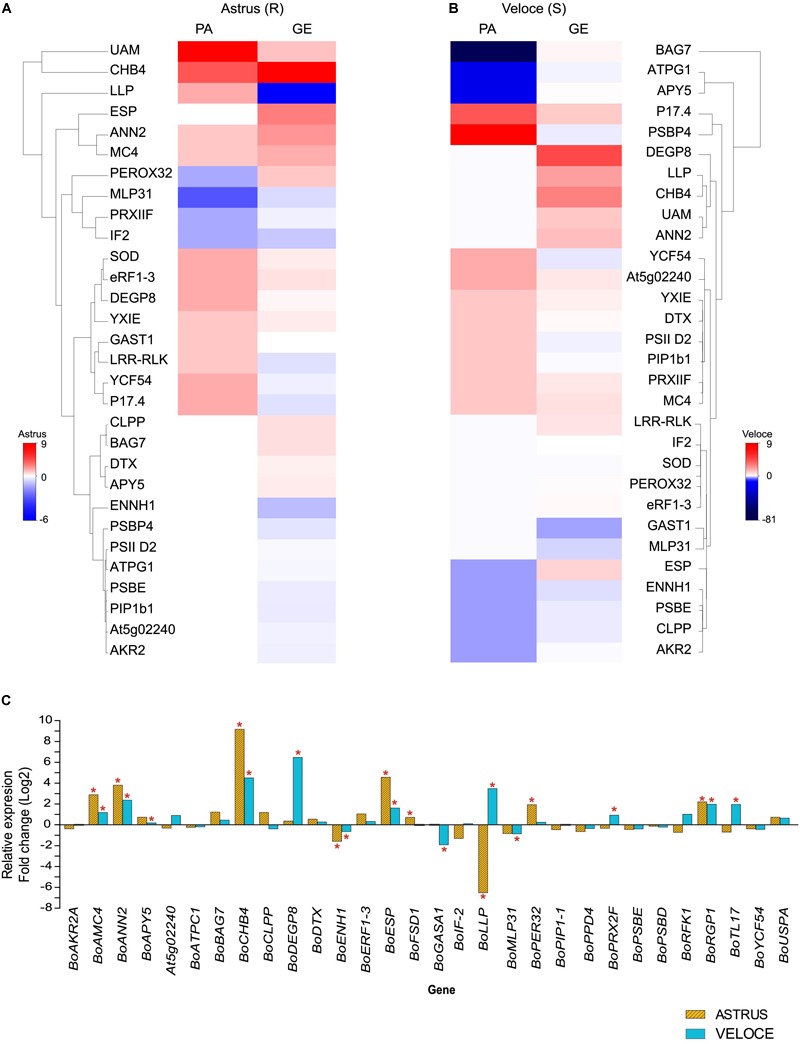
Heatmap showing the correlation between protein abundance (PA) and gene expression (GE) levels in the resistant **(A)** and in the susceptible interaction **(B)**. **(C)** Gene expression of 30 genes in leaves of *B. oleracea* 24 h after inoculation with *X. campestris* pv. *campestris* (Xcc) compared to the control condition. The symbol ^∗^ indicates statistically significant differential expression (*p* ≤ 0.05). The full information of genes and gene products are presented in Table 1. *Bo, Brassica oleracea* gene name homologous to *A. thaliana*.

In this work, we also analyzed the gene expression levels by qRT-PCR of 30 selected genes encoding the differential proteins identified (Figure 2 and Table 1), based on biological process (defense-related), fold-change (≥1.5 increased or decreased in both cultivars) and previous studies (Villeth et al., 2016; Ribeiro et al., 2018). As expected, the expression levels of many mRNAs did not correlate with protein abundance and different clusters could be observed in the heatmap generated to compare these levels (Figure 2). In the resistant cultivar, among the 14 proteins showing increased abundance (statistically validated), 5 corresponding genes showed upregulation (*BoAMC4; BoANNA2; BoCHB4; BoRGP1; BoFSD1*), and among the 4 proteins showing decreased abundance, 1 corresponding gene showed downregulation (*BoPER32*) and the others were not statistically significant. Similar results were obtained for the susceptible cultivar: 10 proteins showed increased abundance, out of which 4 corresponding genes were upregulated, while 8 proteins showed decreased abundance and 2 corresponding genes showed downregulation (*BoENH1* and *BoPRX2F*).

**Table 1:** Differential proteins and encoding genes analyzed by qRT-PCR analysis (RI:RC and SI:SC interactions) and discussed in the proposed interaction model.

Gene^1^	Gene product (full name)	Protein (SN)^2^	UniProt Accession #	Protein Fold change		Gene Fold change (log_2_)		Gene ontology (biological process)^3^
			
Differential genes analyzed by qRT-PCR and included in the model				R	S	R	S	
*BoAKR2A*	Ankyrin repeat domain-containing protein 2-like	AKR2	A0A0D3BK51	ni	-2	-0.37	-0.003	Protein targeting to chloroplast
*BoAMC4*	Metacaspase-4	MC4	A0A0D3D1T5^4^	2	2	2.88^∗^	1.172^∗^	Positive regulation of programmed cell death; protein autoprocessing
*BoANN2*	Annexin	ANN2	I3Y171	2	ni	3.80^∗^	2.357^∗^	Calcium ion transmembrane transport; response to oxidative stress
*BoAPY5*	Apyrase 5	APY5	A0A0D3CA22	nd	-10	0.73	0.179	None predicted
At5g02240	Uncharacterized protein At5g02240	At5g02240	A0A0D3EID2^4^	nd	3	-0.31	0.897^∗^	Response to abscisic acid
*BoATPC1*	ATP synthase gamma chain 1, chloroplastic	ATPG1	A0A0D3E873	nd	-11	-0.23	-0.188	ATP synthesis coupled proton transport
*BoBAG7*	BAG family molecular chaperone regulator 7-like	BAG7	A0A0D3A4W0	ni	-81	1.21	0.452	Cellular response to unfolded protein; protein folding; cellular response to heat
*BoCHB4*	Basic endochitinase CHB4-like	CHB4	A0A0D3BPL2	6	ni	9.17^∗^	4.495^∗^	Cell wall macromolecule catabolic process; chitin catabolic process; response to virus; systemic acquired resistance
*BoCLPP*	ATP-dependent Clp protease proteolytic subunit	CLPP	A0A0D3A7F3	ni	-2	1.18	-0.385	Protein quality control for misfolded or incompletely synthesized proteins
*BoDEGP8*	Protease do-like 8, chloroplastic	DEGP8	A0A0D3BZW4^4^	3	ni	0.35	6.457^∗^	Photosystem II repair
*BoDTX*	Protein detoxification	DTX	A0A0D3BJ77	ni	2	0.54	0.268	Abscisic acid transport; drug transmembrane transport; regulation of response to water deprivation
*BoENH1*	Rubredoxin_like, 1	ENNH1	A0A0D3AJG1	nd	-2	-1.58^∗^	-0.639^∗^	None predicted
*BoERF1-3*	Eukaryotic peptide chain release factor subunit 1–3	eRF1-3	A0A0D3E0E3^4^	3	nd	1.05	0.317	Cytoplasmic translational termination; regulation of growth; translational termination
*BoESP*	Epithiospecifier-like	ESP	A0A0D3CQU9	nd	-2	4.56^∗^	1.607^∗^	Defense response to bacterium; catabolic process; nitrile biosynthetic process; response to jasmonic acid
*BoFSD1*	Superoxide dismutase	SOD	F8U7Z7	3	nd	0.71^∗^	-0.017	Cellular response to oxidative stress; defense response to bacterium; cellular response to salt stress; cellular response to UV-B
*BoGASA1*	Gibberellin-regulated protein 1	GAST1	A0A0D3D0Y9^4^	2	nd	0.01	-1.905^∗^	Response to abscisic acid; response to brassinosteroid; response to gibberellin
*BoIF-2*	Translation initiation factor IF-2	IF2	A0A0D3CAZ7	-2	nd	-1.30	0.110	Translational initiation; translation; nucleotide binding
*BoLLP*	Lectin-like protein At3g16530	LLP	A0A0D3CJY3	3	ni	-6.51^∗^	3.470^∗^	Response to chitin; response to oomycetes
*BoMLP31*	MLP-like protein 31	MLP31	A0A0D3APR4	-4	ni	-0.83	-0.860^∗^	Defense response
*BoPER32*	Peroxidase 32	PEROX32	A0A0D3E2V6	-2	nd	1.92^∗^	0.239	Response to oxidative stress; hydrogen peroxide catabolic process; response to cytokinin
*BoPIP1-1*	Aquaporin PIP1b1	PIP1b1	Q9FUL1^4^	ni	2	-0.46	-0.003	Water transport; response to water deprivation
*BoPPD4*	psbP domain-containing protein 4, chloroplastic	PSBP4	A0A0D3D1B1^4^	nd	9	-0.64	-0.349	Photosynthesis
*BoPRX2F*	Peroxiredoxin IIF, mitochondrial	PRXIIF	A0A0D3CMF0^4^	-2	2	-0.33	0.920^∗^	Cell redox homeostasis; response to cadmium ion; response to oxidative stress
*BoPSBE*	Cytochrome b559 subunit alpha	PSBE	A0A0H3Y313^4^	nd	-2	-0.44	-0.383	Photosynthetic electron transport chain
*BoPSBD*	Photosystem II D2 protein	PSII D2	A0A191SEU8	nd	2	-0.13	-0.223	Photosynthetic electron transport in photosystem II; protein-chromophore linkage
*BoRFK1*	LRR receptor-like serine/threonine-protein kinase At1g29720	LRR-RLK	A0A0D3CSX5	2	nd	-0.71	1.010	Protein autophosphorylation; regulation of innate immune response; jasmonic acid and ethylene-dependent systemic resistance
*BoRGP1*	UDP-arabinopyranose mutase 1-like	UAM	A0A0D3B9D8	9	ni	2.20^∗^	1.968^∗^	Plant-type cell wall organization or biogenesis
*BoTL17*	Thylakoid lumenal 17.4 kDa protein, chloroplastic	P17.4	A0A0D3EAP2^4^	3	6	-0.69	1.944^∗^	Protein binding
*BoYCF54*	Uncharacterized protein Ycf54	YCF54	A0A0D3ECB6^4^	3	3	-0.38	-0.436	None predicted
*BoUSPA*	Universal stress protein YxiE-like	YXIE	A0A0D3CTQ3	2	2	0.73	0.642	None predicted

**Gene^1^**	**Gene product (full name)**	**Protein (SN)^2^**	**UniProt Accession #**	**Protein Fold change**		**Gene ontology (biological process)^3^**		
			
**Additional proteins included in the model**				**R**	**S**			

*BoTRXM*	Thioredoxin M chloroplastic	TRXM	A0A0D3DZ53^4^	2	2	Cell redox homeostasis; glycerol ether metabolic process; regulation of carbohydrate metabolic process		
*BoPRXQ*	Peroxiredoxin Q, chloroplastic isoform X2	PRXQ	A0A0D2ZRQ6^4^	2	nd	Cell redox homeostasis		
*BoPRX*	Peroxiredoxin- Chloroplastic	PRX	A0A0D3BYD5^4^	2	nd	Cell redox homeostasis		
			A0A0D3DSN3^4^	2	nd			
*106329510*	Pectinesterase	PEM17	A0A0D3B6U2^4^	4	2	Cell wall modification; pectin catabolic process		
*BoGSTU5*	Glutathione *S*-transferase U5	GSTU5	A0A0D3B771	2	nd	Response to oxidative stress; response to toxic substance; toxin catabolic process		
*BoGSTU19*	Glutathione *S*-transferase U19	GSTU19	A0A0D3CVZ5	2	-2	Glutathione metabolic process; response to oxidative stress; cellular response to water deprivation		
*BoBoAIG2*	Aig2 protein	AIG2	A0A0D3BZV5^4^	2	2	Response to bacterium		
*106337169*	Ferredoxin	FDX	A0A0D3BV84^4^	2		Transport; electron transport		
*BoLFNR*	Ferredoxin–Nadp leaf isozyme 1 chloroplastic	FNR	A0A0D3E2R^4^	1.5	nd	Defense response to; defense response to fungus, incompatible interaction; photosynthesis; response to cytokinin		
*BoLFNR2*	Ferredoxin–Nadp leaf isozyme 2 chloroplastic	FNR2	A0A0D3DQI2^4^	2	nd	Defense response to; defense response to fungus, incompatible interaction; photosynthesis; response to cytokinin		
*BoGF14 BoKAPPA*	14-3-3 GF14 kappa isoform X1	GF14 kappa	A0A0D3BET0	2	nd	Regulation of metabolic; response to freezing		
*BoAKR4C8*	Aldo-keto reductase family 4 member C8	AKR4C8	A0A0D3BR44	2	-2	Oxidation-reduction process; response to cadmium ion; response to toxic substance; response to cold; response to salt stress		
*BoPBH2*	Prohibitin 2, mitochondrial-like	mtPBH2	A0A0D3C7E7^4^	2	2	Mitochondrion organization; cell division; defense response to bacterium; negative regulation of cell division; response to auxin		
*BoVDAC4*	Mitochondrial outer membrane porin 4	mtVDAC4	A0A0D3B2Z9^4^	2	ni	Regulation of growth; response to bacterium		
*BoMDH*	Malate dehydrogenase mitochondrial	mtMDH	A0A0D3CQE1^4^	3	2	Carbohydrate metabolic process; malate metabolic process; tricarboxylic acid		
			A0A0D3CQN2^4^	2	2	cycle		
			A0A0D3BMU9	4	-2			
*BoMDH*	Malate dehydrogenase, chloroplastic	chlMDH	A0A0D3CGY3	2	1.5	Carbohydrate metabolic process; malate metabolic process; tricarboxylic acid cycle		
*106341843*	Glucan endo-1,3-beta-glucosidase-like	BG	A0A0D3BXB6	7	3	Carbohydrate metabolic process		
*106300472*	Glucan endo-1,3-beta-glucosidase-like (beta-1,3-glucanase)	BG_ppap	A0A0D3CTF1^4^	2	ni	Carbohydrate metabolic process; cell communication		
*BoGAPDH*	Glyceraldehyde-3-phosphate dehydrogenase, chloroplastic	chlGAPDH	A0A0D3DXN8	2	nd	Glucose metabolic process		
*BoPAP 1*	Plastid-lipid-associated 1, chloroplastic	chlPAP 1	A0A0D3E8B6^4^	2	2	Photoinhibition; response to abscisic acid; response to cold		
			A0A0D3E8B7^4^	2	2			
			A0A0D3B8J8	2	3			
*BoPAP 2*	Plastid-lipid-associated 2	PAP 2	A0A0D3A546	2	nd	None predicted		
*BoPAP 3*	Plastid-lipid-associated 3	PAP 3	A0A0D3BRT9^4^	2	2	None predicted		
*BoUSPA*	Universal stress A	USP-A	A0A0D3CN30^4^	2	ni	None predicted		
*BoPEPR1*	Leucine-rich repeat receptor kinase Pepr1	PEP1	A0A0D3D099^4^	3	2	Immune response; innate immune response; response to jasmonic acid; response to wounding		
*NA*	Leucine-rich repeat receptor-like serine threonine- kinase At3g14840	LRR-RLK	A0A0D3BBD1^4^	2	ni	Protein autophosphorylation; regulation of innate immune response; jasmonic acid and ethylene-dependent systemic resistance		
*BoPTI12*	PTI1-like tyrosine-protein kinase 2	PTI1-2	A0A0D3B4A1	1.5	nd	Defense response; protein phosphorylation		
*BoGAPC*	Glyceraldehyde-3-phosphate dehydrogenase	GAPDH	A0A0D2ZPE9	-2	nd	Glucose metabolic process		
			A0A0D3C9I2^4^	-2	nd			
*106335373*	Malate dehydrogenase [NADP] chloroplastic-like	chlMDH	A0A0D3B2U9	-2	nd	Carbohydrate metabolic process; malate metabolic process		
*BoGPX1*	Glutathione peroxidase mitochondrial	mtGPX	A0A0D3AT05	-2	nd	Response to oxidative stress		
*BoDHAR1*	Glutathione *S*-transferase DHAR1, mitochondrial-like	mtDHAR	A0A0D3DQE3	-2	nd	Cellular response to hydrogen peroxide; defense response; positive regulation of salicylic acid mediated signaling pathway; response to jasmonic acid		
*BoPER3*	Peroxidase 3-like	PEROX3	A0A0D3C7R9^4^	-2	nd	Hydrogen peroxide catabolic process; response to oxidative stress		
*BoPER32*	Peroxidase 32	PER32	A0A0D3E2V6	-2	nd	Hydrogen peroxide catabolic process; response to cytokinin; response to oxidative stress		
*BoLHCB5*	Chlorophyll a-b binding protein CP26, chloroplastic	CP26	A0A0D3B7Z5	-4	ni	Non-photochemical quenching; photosynthesis, light harvesting; photosystem II assembly; protein-chromophore linkage		
*BoLHCB4.2*	Chlorophyll a-b binding protein CP29.2, chloroplastic	CP29.2	A0A0D3CLT0	-2	2	Photosynthesis, light harvesting; protein-chromophore linkage; response to blue light; response to cytokinin		
*NA*	Chlorophyll a-b binding protein CP43, chloroplastic	CP43	A0A0D3CFB6	-2	ni	Photosynthetic electron transport in photosystem II; protein-chromophore linkage		
*BoRPN*	26S proteasome non-ATPase regulatory subunit 5	RPN5	A0A0D3CJZ8	-2	nd	Proteasome assembly; translation		
*BoRPT3*	26S protease regulatory subunit 6B homolog	26Sp6B	A0A0D3AL95	2	ni	Protein catabolic process		
*BoUBC36*	Ubiquitin-conjugating enzyme E2 36	UBC36	A0A0D3ARJ5	-2	nd	Postreplication repair; protein K63-linked ubiquitination		
*BoUBC7*	Ubiquitin-conjugating enzyme E2 7	UBC7	A0A0D3ECQ3	-2	nd	Protein ubiquitination		

*^*1*^ Brassica oleracea (*Bo*) gene name based on the homolog in *Arabidopsis thaliana.* “N/A” refers to non-annotated. ^*2*^ Short name of gene product. nd, non-differential protein (fold change < 1.5); ni, non-identified proteins in one of the cultivars. The positive numbers indicate gene fold change up-regulated or increased abundance of gene product and the negative down-regulated or decreased abundance. Log_*2*_, results presentation of fold change values for gene relative expression evaluated by qRT-PCR. R, resistant; S, susceptible. ^*3*^ Gene ontology, summering the principal biological process terms showed in database; mt, mitochondrial; chl, chloroplastic, prefix used here for differentiation of gene product localization. ^*4*^ Gene product identified in enrichment chloroplast simple. ^∗^Differential gene expression statistically validated (*p* ≤ 0.05).*

The differences observed between protein abundance and gene expression levels has been widely reported and may be explained by the regulatory processes that can occur after mRNA transcription, including post-transcriptional, translational, post-translational and protein degradation regulation mechanisms, as well as half-life of RNA and of the corresponding proteins (de Sousa Abreu et al., 2009; Lee et al., 2011; Vogel and Marcotte, 2012).

### Xcc-Responsive Proteins in the Resistant and Susceptible Interaction

The proteome analysis of the resistant cultivar (RI:RC) revealed 338 differentially abundant proteins (215 increased and 123 decreased) while in the susceptible cultivar comparison (SI:SC) 361 differential proteins (225 increased and 136 decreased) were detected. The GO analysis revealed the same over-represented GO terms in both resistant and susceptible interactions (Supplementary Table S3), including cell metabolism, protein biosynthesis, processing and degradation, photosynthesis, disease/defense response and uncharacterized proteins (proteins with no GO information).

A higher number of energy metabolism proteins (Supplementary Table S3) were identified in the susceptible cultivar, most of which showed decreased abundance. It is known that there is intense activity of the main glycolytic pathways during plant–pathogen interaction and alterations in sugar metabolism in the host plant can be crucial for pathogen control, since both organisms compete for nutrients (reviewed by Kanwar and Jha, 2019). It is important to highlight that most of the differential proteins related to energy metabolism were detected only in chloroplast enriched samples, which reinforces the importance of analyzing organelle enriched samples to get a better picture of the plant–pathogen interaction. It is known that photosynthesis is severely affected during biotic and abiotic stresses since resistance has an energy cost. Although the molecular participation of chloroplast in plant immunity is not clear, it has been shown that chloroplasts can have a crucial role in the plant basal immune system that involves PAMPs signaling, Ca^2+^ signaling pathways, as well as salicylic and jasmonic acid (JA) production (Grant and Jones, 2009; Padmanabhan and Dinesh-Kumar, 2010; Nomura et al., 2012).

### Energy Metabolism Proteins

Three malate dehydrogenase mitochondrial proteins (A0A0D3CQE1; A0A0D3CQN2) showed increased abundance in both cultivars, however, one of them (A0A0D3BMU9) showed increased abundance in the resistant cultivar (4-fold) and decreased abundance in the susceptible one (14-fold), when compared to the control condition. Malate is implicated in many plant metabolic processes, including TCA cycle, Calvin cycle, and in pH regulation and ion transport in roots. Malate dehydrogenase, an important malate metabolizing enzyme, has been associated with plant defense, suggesting that the increased abundance of this enzyme can provide resources for biosynthesis of defense compounds (Rhodes et al., 1968; Walter et al., 1988; Casati et al., 1999). In a previous study, one MDH showed increased abundance in brassica–Xcc resistant interaction and was associated with the activation of photosynthetic metabolism (Villeth et al., 2016). Other metabolism proteins such as fructose-1,6-biphosphate, cytosolic EC 3.1.3.11 (A0A0D3BSL1), basic endochitinase CHI-B4-like, EC 3.2.1.14 (A0A0D3B6J8; A0A0D3BPL2) and UDP-arabinopyranose mutase 1-like (A0A0D3B9D8), were increased in RI (9-, 12-, 9- and 6-fold, respectively) and the first two (A0A0D3BSL1 and A0A0D3B6J8) were decreased in SI (28- and 12-fold, respectively), while UDP-arabinopyranose level was unchanged in SI. In the analysis of gene expression (Figure 2 and Table 1), *BoCHI-4 like* and *BoRGP1* were upregulated, with a higher expression in RI:RC (578 and fivefold, respectively) when compared to SI:SC (23- and 4-fold, respectively), suggesting that these proteins, besides being involved in energy metabolism, can have an important role in plant defense. Based on the proteomic and qRT-PCR results, the basic *endochitinase BoCHI-B4-like* gene (GAQY01039586.1) was selected for overexpression in the model plant *A. thaliana* for functional validation.

The metabolic pathways role in defense response process is not well understood, however, our results were consistent with other studies, which suggest that the positive regulation of metabolism can initiate a signaling cascade in the signal transduction pathway, leading to a defense response (Rojas et al., 2014). On the other hand, the pathogen can acquire metabolites from the host cell and the plant can respond to prevent the loss of metabolites by increasing the uptake of monosaccharides, limiting the available extracellular sugar for bacteria. This could be a strategical antimicrobial response, since this competitive reaction can lead to the restriction of the delivery of virulence factors (Vogel and Marcotte, 2012; Couto and Zipfel, 2016; Yamada et al., 2016). Indeed, in *Brassica* and *Arabidopsis* it has been demonstrated that sugar transporters, such as SWEET transporters that mediate sugar export are positively regulated upon pathogen infection (Chen et al., 2010, 2012; Jian et al., 2016), which could indicate co-evolution for nutrient competition during plant–pathogen interaction (Chen et al., 2010). Studies have suggested that *Xanthomonas* effectors can recognize SWEET proteins from the plant and induce sugar export from the cell to be used as carbon source for bacterial growth (Cohn et al., 2014; Huang et al., 2016).

### Proteins Involved in Photosynthesis and Protein Biosynthesis, Processing and Degradation

As expected, several photosynthesis-related proteins were differentially abundant in both interactions, such as photosystem II CP43 reaction center protein, chlorophyll a-b binding protein CP29.2 and CP26, Ribulose bisphosphate carboxylase (Supplementary Table S3). Most proteins related to photosynthesis in the resistant interaction showed decreased abundance (18%), which is consistent with previous results obtained by Ribeiro et al. (2018), when analyzing the same cultivar at the same time point by 2-DE. On the other hand, in this study, most photosynthesis-related proteins were increased in the SI:SC interaction. In this study, we used chloroplast enriched samples and observed that several of the proteins with increased abundance were detected in chloroplasts samples (Supplementary Table S3), which once again reinforce the importance of subproteome analysis to better understand the global protein interaction profile.

In this study, a clear imbalance in metabolic and photosynthetic processes in both cultivars could be observed, however, it is possible that the resistant plant may have a higher recovery capacity than the susceptible plant, since homeostasis and repair proteins were more abundant in the resistant interaction than in the susceptible. It is known that the impaired metabolic capacity can directly influence the functioning of the photosynthetic apparatus (Raven et al., 2007), correlating metabolic alterations with response to pathogens.

Another over-represented GO term observed in this study was protein biosynthesis, protein processing and degradation (folding, assembly, fate and degradation). Proteins mainly involved in transcription, translation, post-translational and transduction processes were observed with increased abundance in both interactions, including several ribosomal proteins (30S, 40S, and 50S in RI:RC; 50S and 60S in SI:SC). It is noteworthy that the BAG (Bcl-2 associated athanogene) family molecular chaperone regulator 7-like (A0A0D3A4W0) showed a pronounced decreased abundance (81-fold) in SI compared to the control, and in resistant plants this protein was not detected. qRT-PCR results showed an upregulation trend in RI and downregulation trend in SI (Figure 2). These results suggest post-transcriptional or post-translational regulation events, since mRNA and protein levels were highly distinct. The BAG7 belongs to Class III of BAG family proteins, which is composed by eight proteins encoded by highly conserved genes, widely distributed in living organisms (Weissbach et al., 1994; Takayama et al., 1995). In plants, BAG proteins have been considered multifunctional and known to regulate the cytoprotective process during biotic and abiotic stresses (Doukhanina et al., 2006). Li et al. (2016) identified proteins of this family that confer resistance in *Arabidopsis* against the fungal pathogen *Botrytis cinerea*, showing evidence of the participation of BAG proteins in innate immunity processes.

### Disease/Defense Response Proteins

Among the disease/defense response proteins identified in our study, most were increased in both cultivars and were involved with oxidative stress (Supplementary Figure S4). However, a higher number of pathogen-related proteins associated with plant responses showed increased abundance in the resistant cultivar (16%), whereas only a few (5%) were increased in the susceptible cultivar. The increased defense proteins identified in both cultivars were annexin (I3Y171), AIG2 (A0A0D3BZV5), ferredoxin (A0A0D3BV84), ferredoxin-NADP leaf isozyme 1 and 2 chloroplastic (A0A0D3E2R1; A0A0D3DQI2) and mitochondrial outer membrane protein 4 (A0A0D3B2Z9).

Several increased proteins in susceptible plants were involved with responses to abscisic acid (ABA), while in resistant plants these proteins showed decreased abundance. ABA is a phytohormone, known as a signaling molecule, responsible for the regulation of abiotic stress response (Taiz et al., 2017). Studies have shown that ABA can suppress the plant immune response, (Kim et al., 2011; Desclos-Theveniau et al., 2012) and in many pathosystems, this phytohormone can act antagonistically to the salicylic acid (SA) pathway. SA is another important phytohormone that can confer plant resistance against pathogens (Audenaert et al., 2002; Jiang et al., 2010). Furthermore, ABA can suppress the MAPK pathway, causing immunosuppression in *A. thaliana* and possibly in other cruciferous plants (Mine et al., 2017). ABA’s effect during plant–pathogen interaction is considered complex, however, it is possible that the increased abundance of proteins involved with ABA response can be a mechanism, which can favor susceptibility (Kim et al., 2011; Desclos-Theveniau et al., 2012).

The increase of ABA can also lead to the accumulation of other proteins such as aquaporins. In this study, the aquaporins PIP3 (Q9FUC0) and *Bo*PIP1b1 (Q9FUL1) were differentially abundant; the second was evaluated by qRT-PCR and showed a downregulation trend in both cultivars. These aquaporins were identified in chloroplast samples with increased abundance only in the susceptible plants. PIP aquaporins are intramembrane channels important for the transport of water and CO_2_ in the plant tissues (Luu and Maurel, 2005; Verkman, 2013). The detection of these proteins in chloroplast samples was not expected, however, since we sampled intact and broken chloroplasts, it is possible that some non-chloroplastic proteins were also isolated. Our results suggest that the accumulation of these proteins may be related to ABA, as observed by Aroca et al. (2006), in leaves of *Phaseolus vulgaris* after ABA treatment. Aquaporins are multifunctional and some isoforms are able to detect pathogen molecular patterns (PAMPs) such as harpins (Zhu et al., 2000; Flexas et al., 2007). The transport of hydrogen peroxide has also been associated to aquaporins (Taiz et al., 2017). It was demonstrated that the loss of function of the gene locus *AtPIP1;4* in *Arabidopsis* cancels the import of apoplastic H_2_O_2_ induced by the pathogen and consequently blocks the plant immune response (Tian et al., 2016).

Curiously, another protein named epithiospecifier-like (ESP-like; A0A0D3CQU9) involved in defense response, was decreased in susceptible plants and unchanged in resistant plants. Conversely, the expression of *ESP* gene was upregulated according to qRT-PCR results in both cultivars (23-fold change in the resistant plant and 3-fold change in the susceptible). In the previous study performed by Ribeiro et al. (2018), another resistant cabbage cultivar (União) was analyzed and ESP protein was exclusively identified in the resistant cultivar infected with Xcc by 2-DE analysis, demonstrating that the regulation of ESP protein can be important for the plant defense against Xcc. The ESP protein is related to the glucosinolate pathway involved in plant protection against herbivory pests. Glucosinolates are secondary metabolites, known as phytoanticipins (preformed antimicrobial compounds), representing one of the first chemical barriers against pathogen attack (Osbourn, 1996). These metabolites can be found extensively in *Brassicaceae* plants (i.e., broccoli, cabbage, mustard), and are biologically active compounds reported in some processes of plant defense including stress response and antioxidant activities (Bennett and Wallsgrove, 1994; Halkier and Gershenzon, 2006).

### Interaction Model of Resistant *B. oleracea*–Xcc Interaction

**FIGURE 3:**
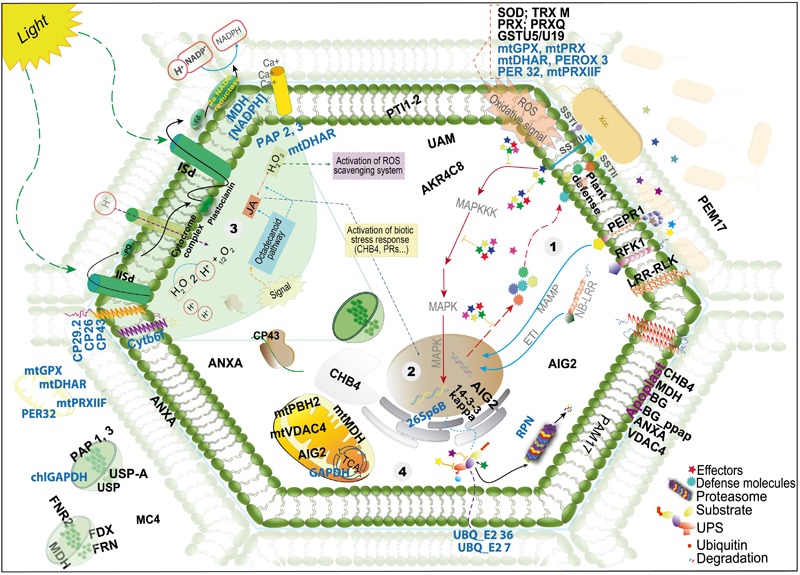
Schematic view of a model proposed with the proteins identified in the resistant *Brassica oleracea*–*Xanthomonas campestris* pv. *campestris* (Xcc) infected leaf and chloroplast-enriched proteomes. The figure shows the localization, as proposed by UniProt database; detailed information on the proteins is presented in Table 1. The names in black and blue indicate proteins with increased and decreased abundance, respectively. The steps begin at the recognition of the pathogen, involving important signaling proteins, activation of molecular defense response pathways and oxidative stress response (steps 1–3), followed by UPS (ubiquitin pathway system) modulation and repair proteins (step 4), as well as alteration of metabolic and photosynthetic pathways (steps 3 and 4).

Overall, in this study, we observed that the protein profiles of the resistant and susceptible plants were similar, especially regarding the predominant GO terms. However, a higher number of pathogen-related proteins were identified in the resistant plants and therefore we propose a model of this interaction based on protein localization and their role in the cell (Figure 3 and Table 1). This model can help better understand the plant response to Xcc infection and provide candidate genes for the development of more efficient pathogen control strategies.

The classical mechanism of bacterial recognition occurs at the beginning of infection, in an attempt to neutralize the effectors released by the bacterium and repair the damage caused in the cell. In the resistant cultivar Astrus, we found several proteins with increased abundance probably involved in this initial response (see step 1 in the model), such as Lectin-like protein (A0A0D3CJY3), leucine-rich repeat receptor kinase (A0A0D3D099), leucine-rich repeat receptor-like serine threonine- kinase (A0A0D3CSX5). These transmembrane signaling proteins, together with other proteins, such as NB-LRR, can be essential to sense the pathogen and promote systemic immunity (O’Neill and Bowie, 2007; Couto and Zipfel, 2016). Other signaling proteins identified showing increased abundance were the universal stress protein A (A0A0D3CN30) and universal stress PHOS34-like (A0A0D3CTQ3). Although the exact role of PHOS34 in plant defense is not known, studies have reported that this protein can be phosphorylated by MPK3 and MPK6, and after treatment with the flagellin flg22 peptide (Merkouropoulos et al., 2008), suggesting that this protein may be related with cellular signaling in the presence of the bacterium.

Several antioxidant/detoxification proteins were also increased, including superoxide dismutase Fe (F8U7Z7), peroxidase (A0A0D3C7R8), peroxiredoxin (A0A0D3DSN3; A0A0D2ZRQ6; A0A0D3BYD5) and glutathione S-transferase U5 and U19 (A0A0D3B771; A0A0D3CVZ5). The accumulation of ROS can be toxic to the pathogen by inhibiting and/or reducing its survival (Jones and Dangl, 2006; Zhang and Zhou, 2010). However, ROS accumulation can lead to the oxidation of important cell components like lipids and genetic material (Sharma et al., 2016). An intriguing result obtained was the decrease of other extra and intracellular antioxidant proteins including glutathione peroxidase mitochondrial; (A0A0D3AT05; A0A0D3DQE3) peroxidase 3-like (A0A0D3C7R9); peroxiredoxin-mitochondrial (A0A0D3CMF0) and peroxidase 32 (A0A0D3E2V6) in the resistant interaction. This result may indicate that a balance in the abundance of proteins related to oxidative stress, maintaining some proteins with increased abundance and others with decreased abundance may be important for an efficient control of the pathogen without extensive damage to the plant tissue.

Once the pathogen overcomes the first line of defense, other events occur in response to the effector delivery into the cell by the type III secretion system. At this stage, proteins such as NB-LRR proteins interact with the pathogen effectors (Spallek et al., 2009; Marino et al., 2012). In this study, we identified a leucine-rich repeat receptor kinase PEPR1 (A0A0D3D099), which has been reported as the receptor for AtPep1, a peptide elicitor from *Arabidopsis* that signals activation of innate immune response against pathogens (Yamaguchi et al., 2006) as well as a probable LRR receptor-like serine/threonine-protein kinase At1g29720 *RFK1-like* (A0A0D3CSX5). Both proteins are integral components of the membrane (model-step 1) and may be involved in triggering a defense response.

Ubiquitination pathway also seems to play an important role in the resistant interaction. The protein 14-3-3-GF14 kappa (A0A0D3BET0), known as a metabolism regulator associated with abiotic stress, was identified and can modulate other proteins by facilitating their degradation by ubiquitins (Fuller et al., 2006; Chang et al., 2009; Liu et al., 2017). The ubiquitin pathway is necessary to tag proteins that should be degraded, however, bacterial effectors may also interact with ubiquitin proteasome system (UPS) as a false system protein (Figure 3). The bacterial effectors can be ubiquitinated and degraded by proteasomes; they can also interfere in the system, act as a ubiquitin ligase or inhibit the specific UPS steps (Collins and Brown, 2010). Proteins related to ubiquitination showed reduced abundance in the present work (A0A0D3CJZ8; A0A0D3ARJ5, A0A0D3ECQ3, A0A0D3BLH4). In a highly resistant plant, ubiquitination proteins also showed reduced abundance at 24 hai (Ribeiro et al., 2018), which may indicate a negative regulation of this pathway, leading to cell death and consequently resulting in limitation of bacterial growth (Spallek et al., 2009; Marino et al., 2012).

Proteins involved with defense against pathogens were also increased including Ferredoxin–NADP leaf isozyme 1 and 2, chloroplastic (A0A0D3E2R1; A0A0D3DQI2) and annexin (I3Y171). Annexins are members of a well-known family of proteins involved in tolerance against environmental stresses and have been studied in tobacco, cotton, *Brassica* and *Arabidopsis* plants (Jami et al., 2008; Konopka-Postupolska et al., 2009; Clark et al., 2012; Szalonek et al., 2015). AIG2 (A0A0D3BZV5) is another defense protein, which has not been functionally characterized yet, however, it is known that the corresponding gene is induced in *Arabidopsis* by the avirulent gene *avrRpt2* of *Pseudomonas syringae* (Reuber and Ausubel, 1996).

Defense response is also highly correlated with the levels of phytohormones, such as JA, ethylene (ET), ABA, and cytokinin. JA is important in the plant defense against various stresses. As seen in the model, the indirect activation of JA by the octadecanoid pathway and H_2_O_2_ accumulation can result in the activation of biotic stress response. The JA pathway can activate other pathways such as the signal transduction pathway, inducing the formation of chemical and physical barriers against pathogen or herbivore attacks (Kazan and Manners, 2008). In addition to the lectin proteins identified, plastid lipid-associated protein 2 and 3, chloroplastic (A0A0D3A546; A0A0D3BRT9) were also identified.

Another phytohormone involved in defense signaling is cytokinin, involved in plant development, cellular differentiation and senescence (Hwang et al., 2012). It has been reported that high levels of this hormone increased plant immunity (Swartzberg et al., 2008; Choi et al., 2010; Argueso et al., 2012). In this study, some proteins responsive to cytokinins were increased including 50S Ribosomal Chloroplastic protein (A0A0D3C0C3); binding partner of ACD11 1 isoform X2 (A0A0D3AJE9) and succinate dehydrogenase subunit 5 mitochondrial-like isoform X2 (A0A0D3DK02). Based on these results, it seems that the regulation of proteins responsive to these phytohormones may play an important role in resistance against Xcc.

Secondary metabolites also play an important role in plant defense. Chloroplastic plastid-lipid-associated proteins (1, 2, and 3), were identified with increased abundance and are associated with the storage of carotenoids in plants and sequestration of hydrophobic compounds (Ting et al., 1998; Laizet et al., 2004; Leitner-Dagan et al., 2006).

Finally, when the initial defense mechanisms are not enough to contain the pathogen, cell death can also be activated. In this study, metacaspase 4 (A0A0D3D1T5), an important protein reported as a participant in the cell death mechanism (Kwon and Hwang, 2013), showed increased abundance in both cultivars. qRT-PCR analysis showed that the corresponding gene was upregulated sevenfold in the resistant cultivar and twofold in the susceptible one. Taken together, the model presented here can represent a step-by-step of the defense mechanism in resistant brassica plants, beginning at the recognition of the pathogen, with the activation of important signaling proteins, molecular defense response pathways, and oxidative stress response (steps 1–3 in the model), followed by UPS modulation and repair proteins (step 4), and alteration of metabolic and photosynthetic pathways (steps 3 and 4). The model may also contribute to better understand the molecular responses during the plant–pathogen interaction reflected by the differential abundance of proteins under Xcc infection.

### Functional Validation by Overexpression of CHI-B4 Like Protein in *A. thaliana*

In this study, several candidate proteins were identified, potentially involved in the resistance response to Xcc. One of these proteins, the CHI-B4 like protein, as mentioned above, showed increased abundance in the proteomic analysis and high gene expression levels. Endochitinase-like proteins, are members of the chitinase family that participate in the catabolic process in the cell (Stintzi et al., 1993). The chitinases are also classified as pathogen related proteins (PRs) and endochitinases belong to group 3 of PRs that cleave chitin molecules. In general, studies have related chitinases to plant–fungus interaction (reviewed by Jalil et al., 2015).

**FIGURE 4:**
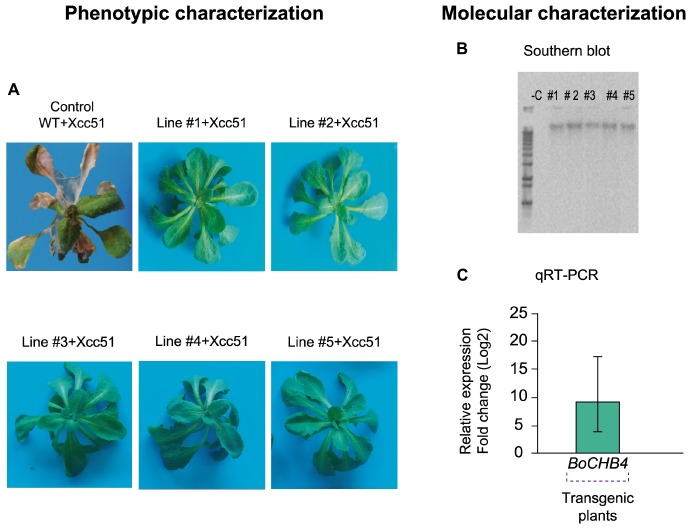
Phenotypic and molecular evaluation of transgenic lines and wild type (WT) *Arabidopsis thaliana* plants. **(A)** *A. thaliana* plants (WT and #1–5 *BoCHB4* lines) inoculated with *Xanthomonas campestris* pv. *campestris* (Xcc 51) at 5 days after inoculation (dai). **(B)** Southern blot analysis of *A. thaliana* transgenic lines (# 1–5) with *BoCHB4* radiolabeled probe. **(C)** Relative expression of *BoCHB4* gene in *A. thaliana* WT and transgenic lines (# 1–5).

*A. thaliana* plants overexpressing cabbage *BoCHB4* gene under the control of CaMV 35S promoter were obtained through transformation. The presence of the transgene was confirmed in five homozygote plants and single copy insertions were observed by Southern blot analysis for each positive event (Figure 4). The transcript level of the transgene was also assessed, and the transgenic lines showed a relative expression level 588-fold higher than the wild type (Col-0). Since *CHI-B4 like* can also be found in *A. thaliana*, an alignment of the nucleotide sequences of both genes (endogenous and transgenic) was performed and showed 84% identity with the *Arabidopsis* gene, and therefore the expression levels detected were probably related to the transgene and not to the endogenous gene.

A phenotypic evaluation of *A. thaliana* plants overexpressing *BoCHB4* was performed and at 48 hai the WT plants began to show the first symptoms (Figure 4). The phenotypic analysis of the WT and transgenic lines after inoculation of Xcc showed that at 5 days after inoculation (dai), among the surviving plants, the WT replicates were almost totally necrotic and most leaves were dead, while the transgenic plants showed no symptoms even at 15 days after inoculation. According to the disease scoring index based on Meyer et al. (2005), *A. thaliana* WT Col-0 was highly susceptible, showing severe necrosis and leaf death, while the transformed plants were highly resistant, since no symptoms were observed. Based on these results, we conclude that the overexpression of *BoCHB4* can confer resistance against the bacterial pathogen Xcc. This work provides an important contribution regarding the comprehension of resistance mechanisms and offers candidate genes to be used in genetic breeding programs aiming at the development of more efficient strategies for black rot disease control.

## Author Contributions

CS performed and designed the experiments, analyzed and interpreted data, and wrote the manuscript. FN, GD, WF, and JJ-N assisted in proteomic data acquisition and analysis. GP, PH, VS, OO-N, and MG-d-S assisted in gene isolation, vector design, and plant transformation. OF analyzed the data and revised the manuscript and AM designed the experiments, wrote the manuscript, and lead the work.

## Conflict of Interest Statement

The authors declare that the research was conducted in the absence of any commercial or financial relationships that could be construed as a potential conflict of interest.

## Footnotes

1 www.uniprot.org

2 https://www.ebi.ac.uk/Tools/msa/clustalo

## Supplementary Material

FIGURE S1 Workflow showing the entire procedure for sample preparation.

FIGURE S2 SDS-PAGE step prior to LC-MS/MS analysis. The section indicated by the red square was excised and submitted to *in gel* digestion using trypsin.

FIGURE S3 Venn diagram showing the total number of proteins identified by the proteomic analysis. **(a)** Quantitative distribution of proteins from inoculated leaves (LRI) and from the control condition (LRC) as well as from chloroplast-enriched inoculated samples (ChlRI) and from the control condition (ChlRC) in the resistant interaction. **(b)** Quantitative distribution of proteins from the susceptible inoculated leaves (LSI) and from the control condition (LSC) as well as from chloroplast-enriched inoculated samples (ChlSI) and from the control condition (ChlSC) in the susceptible interaction.

FIGURE S4 Subclassification of proteins related to disease/defense response, differentially abundant in resistant and susceptible cultivars. **(a)** Proteins with increased and **(b)** decreased abundance in the RI:RC comparison. **(c)** Proteins with increased and **(d)** decreased abundance in the SI:SC comparison. RI, resistant cultivar inoculated; RC, resistant cultivar control; SI, susceptible cultivar inoculated; SC, susceptible cultivar control.

TABLE S1 Primers designed for the genes encoding the identified proteins used in qRT-PCR.

TABLE S2 *Brassica oleracea* var. *capitata* protein data obtained by LC-MS/MS. The data in each cultivar are reported in separate spreadsheets.

TABLE S3 Biological process category of the identified proteins with increased and decreased abundance in both cultivars comparison (RI:RC and SI:SC), as indicated in each spreadsheet.

## References

[B1] Argueso C. T. Ferreira F. J. Epple P. To J. P. C. Hutchison C. E. Schaller G. E. (2012). Two-component elements mediate interactions between cytokinin and salicylic acid in plant immunity. *PLoS Genet.* 8:e1002448. 10.1371/journal.pgen.1002448 22291601 PMC3266875

[B2] Aroca R. Ferrante A. Vernieri P. Chrispeels M. J. (2006). Drought, abscisic acid and transpiration rate effects on the regulation of PIP aquaporin gene expression and abundance in *Phaseolus vulgaris* plants. *Ann. Bot.* 98 1301–1310. 10.1093/aob/mcl219 17028296 PMC2803586

[B3] Audenaert K. De Meyer G. B. Höfte M. M. (2002). Abscisic acid determines basal susceptibility of tomato to *Botrytis cinerea* and suppresses salicylic acid-dependent signaling mechanisms. *Plant Physiol.* 128 491–501. 10.1104/pp.010605 11842153 PMC148912

[B4] Audran C. Serrano I. Rivas S. (2016). Chloroplasts at work during plant innate immunity. *J. Exp. Bot.* 67 3845–3854. 10.1093/jxb/erw088 26994477

[B5] Bayer R. G. Stael S. Teige M. (2015). Chloroplast isolation and affinity chromatography for enrichment of low-abundant proteins in complex proteomes. *Methods Mol. Biol.* 1295 211–223. 10.1007/978-1-4939-2550-6_16 25820724

[B6] Bendahmane A. Farnham G. Moffett P. Baulcombe D. C. (2002). Constitutive gain-of-function mutants in a nucleotide binding site–leucine rich repeat protein encoded at the Rx locus of potato. *Plant J.* 32 195–204. 10.1046/j.1365-313X.2002.01413.x 12383085

[B7] Bennett R. N. Wallsgrove R. M. (1994). Secondary metabolites in plant defence mechanisms. *New Phytol.* 127 617–633. 10.1111/j.1469-8137.1994.tb02968.x 33874382

[B8] Bent A. F. Clough S. J. (1998). “*Agrobacterium* germ-line transformation: transformation of *Arabidopsis* without tissue culture,” in *Plant Molecular Biology Manual* eds Gelvin S. B. Schilperoort R. A. (Dordrecht: Springer) 17–30.

[B9] Camargo L. E. A. Williams P. H. Osborn T. C. (1995). Mapping of quantitative trait loci controlling resistance of *Brassica oleracea* to *Xanthomonas campestris* pv. *campestris* in the field and greenhouse. *Phytopathology* 85 1296–1300. 10.1094/Phyto-85-1296

[B10] Casati P. Drincovich M. F. Edwards G. E. Andreo C. S. (1999). Malate metabolism by NADP-malic enzyme in plant defense. *Photosynth. Res.* 61 99–105. 10.1023/A:1006209003096

[B11] Chang I. F. Curran A. Woolsey R. Quilici D. Cushman J. C. Mittler R. (2009). Proteomic profiling of tandem affinity purified 14-3-3 protein complexes in *Arabidopsis thaliana*. *Proteomics* 9 2967–2985. 10.1002/pmic.200800445 19452453 PMC4077669

[B12] Chen L.-Q. Hou B.-H. Lalonde S. Takanaga H. Hartung M. L. Qu X.-Q. (2010). Sugar transporters for intercellular exchange and nutrition of pathogens. *Nature* 468 527–532. 10.1038/nature09606 21107422 PMC3000469

[B13] Chen L.-Q. Qu X.-Q. Hou B.-H. Sosso D. Osorio S. Fernie A. R. (2012). Sucrose efflux mediated by SWEET proteins as a key step for phloem transport. *Science* 335 207–211. 10.1126/science.1213351 22157085

[B14] Choi J. Huh S. U. Kojima M. Sakakibara H. Paek K.-H. Hwang I. (2010). The cytokinin-activated transcription factor ARR2 promotes plant immunity via TGA3/NPR1-dependent salicylic acid signaling in *Arabidopsis*. *Dev. Cell* 19 284–295. 10.1016/j.devcel.2010.07.011 20708590

[B15] Clark G. B. Morgan R. O. Fernandez M. P. Roux S. J. (2012). Evolutionary adaptation of plant annexins has diversified their molecular structures, interactions and functional roles. *New Phytol.* 196 695–712. 10.1111/j.1469-8137.2012.04308.x 22994944

[B16] Cohn M. Bart R. S. Shybut M. Dahlbeck D. Gomez M. Morbitzer R. (2014). *Xanthomonas axonopodis* virulence is promoted by a transcription activator-like effector–mediated induction of a SWEET sugar transporter in cassava. *Mol. Plant Microbe Interact.* 27 1186–1198. 10.1094/MPMI-06-14-0161-R 25083909

[B17] Collins C. A. Brown E. J. (2010). Cytosol as battleground: ubiquitin as a weapon for both host and pathogen. *Trends Cell Biol.* 20 205–213. 10.1016/j.tcb.2010.01.002 20129784

[B18] Corthals G. L. Wasinger V. C. Hochstrasser D. F. Sanchez J. C. (2000). The dynamic range of protein expression: a challenge for proteomic research. *Electrophoresis* 21 1104–1115. 10.1002/(SICI)1522-2683(20000401)21:6<1104::AID-ELPS1104>3.0.CO;2-C 10786884

[B19] Couto D. Zipfel C. (2016). Regulation of pattern recognition receptor signalling in plants. *Nat. Rev. Immunol.* 16 537–552. 10.1038/nri.2016.77 27477127

[B20] de Sousa Abreu R. Penalva L. O. Marcotte E. M. Vogel C. (2009). Global signatures of protein and mRNA expression levels. *Mol. Biosyst.* 5 1512–1526. 10.1039/b908315d 20023718 PMC4089977

[B21] Desclos-Theveniau M. Arnaud D. Huang T.-Y. Lin G. J.-C. Chen W.-Y. Lin Y.-C. (2012). The Arabidopsis lectin receptor kinase LecRK-V.5 represses stomatal immunity induced by *Pseudomonas syringae* pv. *tomato* DC3000. *PLoS Pathog.* 8:e1002513. 10.1371/journal.ppat.1002513 22346749 PMC3276567

[B22] Doukhanina E. V. Chen S. Van Der Zalm E. Godzik A. Reed J. Dickman M. B. (2006). Identification and functional characterization of the BAG protein family in *Arabidopsis thaliana*. *J. Biol. Chem.* 281 18793–18801. 10.1074/jbc.M511794200 16636050

[B23] Etschmann B. Wilcken B. Stoevesand K. Von Der Schulenburg A. Sterner-Kock A. (2006). Selection of reference genes for quantitative real-time PCR analysis in canine mammary tumors using the GeNorm algorithm. *Vet. Pathol.* 43 934–942. 10.1354/vp.43-6-934 17099150

[B24] Flexas J. Ortuño M. F. Ribas-Carbo M. Diaz-Espejo A. Florez-Sarasa I. D. Medrano H. (2007). Mesophyll conductance to CO2 in *Arabidopsis thaliana*. *New Phytol.* 175 501–511. 10.1111/j.1469-8137.2007.02111.x 17635225

[B25] Fuller B. Stevens S. M. Jr. Sehnke P. C. Ferl R. J. (2006). Proteomic analysis of the 14-3-3 family in *Arabidopsis*. *Proteomics* 6 3050–3059. 10.1002/pmic.200500729 16619310

[B26] Grant M. R. Jones J. D. G. (2009). Hormone (dis) harmony moulds plant health and disease. *Science* 324 750–752. 10.1126/science.1173771 19423816

[B27] Guo H. Dickson M. H. Hunter J. E. (1991). *Brassica napus* sources of resistance to black rot in crucifers and inheritance of resistance. *Hortscience* 26 1545–1547. 10.21273/HORTSCI.26.12.1545

[B28] Halkier B. A. Gershenzon J. (2006). Biology and biochemistry of glucosinolates. *Annu. Rev. Plant Biol.* 57 303–333. 10.1146/annurev.arplant.57.032905.105228 16669764

[B29] Han Y. Ma B. I. N. Zhang K. (2005). SPIDER: software for protein identification from sequence tags with de novo sequencing error. *J. Bioinform. Comput. Biol.* 3 697–716. 10.1142/S0219720005001247 16108090

[B30] Huang S. Antony G. Li T. Liu B. Obasa K. Yang B. (2016). The broadly effective recessive resistance gene xa5 of rice is a virulence effector-dependent quantitative trait for bacterial blight. *Plant J.* 86 186–194. 10.1111/tpj.13164 26991395

[B31] Hwang I. Sheen J. Müller B. (2012). Cytokinin signaling networks. *Annu. Rev. Plant Biol.* 63 353–380. 10.1146/annurev-arplant-042811-105503 22554243

[B32] Ignatov A. Kuginuki Y. Hidam K. (2000). Distribution and inheritance of race-specific resistance to *Xanthomonas campestris* pv. *campestris* in *Brassica rapa* and *B. napus*. *J. Russ. Phytopathol. Soc.* 1 89–94.

[B33] Jalil S. U. Mishra M. Ansari M. I. (2015). Current view on chitinase for plant defense. *Trends Biosci.* 8 6733–6743. 28367157

[B34] Jami S. K. Clark G. B. Turlapati S. A. Handley C. Roux S. J. Kirti P. B. (2008). Ectopic expression of an annexin from *Brassica juncea* confers tolerance to abiotic and biotic stress treatments in transgenic tobacco. *Plant Physiol. Biochem.* 46 1019–1030. 10.1016/j.plaphy.2008.07.006 18768323

[B35] Jian H. Lu K. Yang B. Wang T. Zhang L. Zhang A. (2016). Genome-wide analysis and expression profiling of the *SUC* and *SWEET* gene families of sucrose transporters in oilseed rape (*Brassica napus* L.). *Front. Plant Sci.* 7:1464. 10.3389/fpls.2016.01464 27733861 PMC5039336

[B36] Jiang C.-J. Shimono M. Sugano S. Kojima M. Yazawa K. Yoshida R. (2010). Abscisic acid interacts antagonistically with salicylic acid signaling pathway in rice–*Magnaporthe grisea* interaction. *Mol. Plant Microbe Interact.* 23 791–798. 10.1094/MPMI-23-6-0791 20459318

[B37] Jones J. D. G. Dangl J. L. (2006). The plant immune system. *Nature* 444 323–329. 10.1038/nature05286 17108957

[B38] Kamal A. H. M. Kim K.-H. Shin K.-H. Choi J.-S. Baik B.-K. Tsujimoto H. (2010a). Abiotic stress responsive proteins of wheat grain determined using proteomics technique. *Aust. J. Crop Sci.* 4 196–208.

[B39] Kamal A. H. M. Kim K.-H. Shin K.-H. Kim D.-E. Oh M.-W. Choi J.-S. (2010b). Proteomics-based dissection of biotic stress responsive proteins in bread wheat (*Triticum aestivum* L.). *Afr. J. Biotechnol.* 9 7239–7255.

[B40] Kanwar P. Jha G. (2019). Alterations in plant sugar metabolism: signatory of pathogen attack. *Planta* 249 305–318. 10.1007/s00425-018-3018-3 30267150

[B41] Kazan K. Manners J. M. (2008). Jasmonate signaling: toward an integrated view. *Plant Physiol.* 146 1459–1468. 10.1104/pp.107.115717 18390489 PMC2287326

[B42] Kim S. T. Kang K. Y. (2008). “Proteomics in plant defense response,” in *Plant Proteomics: Technologies, Strategies, and Applications* eds Agrawal G. K. Rakwal R. (Hoboken, NJ: John Wiley & Sons, Inc.) 585–604. 10.1002/9780470369630.ch40

[B43] Kim T.-H. Hauser F. Ha T. Xue S. Böhmer M. Nishimura N. (2011). Chemical genetics reveals negative regulation of abscisic acid signaling by a plant immune response pathway. *Curr. Biol.* 21 990–997. 10.1016/j.cub.2011.04.045 21620700 PMC3109272

[B44] Kley J. Heil M. Muck A. Svatoš A. Boland W. (2010). Isolating intact chloroplasts from small *Arabidopsis* samples for proteomic studies. *Anal. Biochem.* 398 198–202. 10.1016/j.ab.2009.11.016 19917261

[B45] Komatsu S. Hossain Z. (2017). *Preface—Plant Proteomic Research.* Basel: Multidisciplinary Digital Publishing Institute. 10.3390/ijms18010088 PMC5297722 28054969

[B46] Konopka-Postupolska D. Clark G. Goch G. Debski J. Floras K. Cantero A. (2009). The role of annexin 1 in drought stress in Arabidopsis. *Plant Physiol.* 150 1394–1410. 10.1104/pp.109.135228 19482919 PMC2705051

[B47] Kwon S. I. Hwang D. J. (2013). Expression analysis of the metacaspase gene family in *Arabidopsis*. *J. Plant Biol.* 56 391–398. 10.1007/s12374-013-0290-4

[B48] Laizet Y. Pontier D. Mache R. Kuntz M. (2004). Subfamily organization and phylogenetic origin of genes encoding plastid lipid-associated proteins of the fibrillin type. *J. Genome Sci. Technol.* 3 19–28. 10.1166/gl.2004.038

[B49] Lee M. V. Topper S. E. Hubler S. L. Hose J. Wenger C. D. Coon J. J. (2011). A dynamic model of proteome changes reveals new roles for transcript alteration in yeast. *Mol. Syst. Biol.* 7:514. 10.1038/msb.2011.48 21772262 PMC3159980

[B50] Leitner-Dagan Y. Ovadis M. Shklarman E. Elad Y. David D. R. Vainstein A. (2006). Expression and functional analyses of the plastid lipid-associated protein CHRC suggest its role in chromoplastogenesis and stress. *Plant Physiol.* 142 233–244. 10.1104/pp.106.082404 16815957 PMC1557619

[B51] Li Y. Kabbage M. Liu W. Dickman M. B. (2016). Aspartyl protease mediated cleavage of BAG6 is necessary for autophagy and fungal resistance in plants. *Plant Cell* 28 233–247. 26739014 10.1105/tpc.15.00626 PMC4746679

[B52] Liu Z. Jia Y. Ding Y. Shi Y. Li Z. Guo Y. (2017). Plasma membrane CRPK1-mediated phosphorylation of 14-3-3 proteins induces their nuclear import to fine-tune CBF signaling during cold response. *Mol. Cell* 66 117–128. 10.1016/j.molcel.2017.02.016 28344081

[B53] Luu D. T. Maurel C. (2005). Aquaporins in a challenging environment: molecular gears for adjusting plant water status. *Plant Cell Environ.* 28 85–96. 10.1111/j.1365-3040.2004.01295.x

[B54] Marino D. Peeters N. Rivas S. (2012). Ubiquitination during plant immune signaling. *Plant Physiol.* 160 15–27. 10.1104/pp.112.199281 22689893 PMC3440193

[B55] Merkouropoulos G. Andreasson E. Hess D. Boller T. Peck S. C. (2008). An *Arabidopsis* protein phosphorylated in response to microbial elicitation, AtPHOS32, is a substrate of MAP kinases 3 and 6. *J. Biol. Chem.* 283 10493–10499. 10.1074/jbc.M800735200 18285339

[B56] Meyer D. Lauber E. Roby D. Arlat M. Kroj T. (2005). Optimization of pathogenicity assays to study the *Arabidopsis thaliana–Xanthomonas campestris* pv. *campestris* pathosystem. *Mol. Plant Pathol.* 6 327–333. 10.1111/j.1364-3703.2005.00287.x 20565661

[B57] Mine A. Berens M. L. Nobori T. Anver S. Fukumoto K. Winkelmüller T. M. (2017). Pathogen exploitation of an abscisic acid-and jasmonate-inducible MAPK phosphatase and its interception by *Arabidopsis* immunity. *Proc. Natl. Acad. Sci.* 114 7456–7461. 10.1073/pnas.1702613114 28652328 PMC5514735

[B58] Mot R. D. Vanderleyden J. (1989). Application of two-dimensional protein analysis for strain fingerprinting and mutant analysis of *Azospirillum* species. *Can. J. Microbiol.* 35 960–967. 10.1139/m89-158

[B59] Nomura H. Komori T. Uemura S. Kanda Y. Shimotani K. Nakai K. (2012). Chloroplast-mediated activation of plant immune signalling in *Arabidopsis*. *Nat. Commun.* 3:926. 10.1038/ncomms1926 22735454

[B60] O’Neill L. A. J. Bowie A. G. (2007). The family of five: TIR-domain-containing adaptors in Toll-like receptor signalling. *Nat. Rev. Immunol.* 7 353–364. 10.1038/nri2079 17457343

[B61] Osbourn A. E. (1996). Preformed antimicrobial compounds and plant defense against fungal attack. *Plant Cell* 8 1821–1831. 10.1105/tpc.8.10.1821 12239364 PMC161317

[B62] Padmanabhan M. S. Dinesh-Kumar S. P. (2010). All hands on deck—the role of chloroplasts, endoplasmic reticulum, and the nucleus in driving plant innate immunity. *Mol. Plant Microbe Interact.* 23 1368–1380. 10.1094/MPMI-05-10-0113 20923348

[B63] Peltier J.-B. Friso G. Kalume D. E. Roepstorff P. Nilsson F. Adamska I. (2000). Proteomics of the chloroplast: systematic identification and targeting analysis of lumenal and peripheral thylakoid proteins. *Plant Cell* 12 319–341. 10.1105/tpc.12.3.319 10715320 PMC139834

[B64] Pfaffl M. W. Horgan G. W. Dempfle L. (2002). Relative expression software tool (REST©) for group-wise comparison and statistical analysis of relative expression results in real-time PCR. *Nucleic Acids Res.* 30:e36 10.1093/nar/30.9.e36 PMC113859 11972351

[B65] Rappsilber J. Mann M. Ishihama Y. (2007). Protocol for micro-purification, enrichment, pre-fractionation and storage of peptides for proteomics using StageTips. *Nat. Protoc.* 2 1896–1906. 10.1038/nprot.2007.261 17703201

[B66] Raven P. H. Evert R. F. Eichhorn S. E. (2007). *Biologia Vegetal, 830.* Rio de Janeiro: Guanabara.

[B67] Reuber T. L. Ausubel F. M. (1996). Isolation of *Arabidopsis* genes that differentiate between resistance responses mediated by the *RPS2* and *RPM1* disease resistance genes. *Plant Cell* 8 241–249. 10.1105/tpc.8.2.241 8742710 PMC161094

[B68] Rhodes M. J. C. Wooltorton L. S. C. Galliard T. Hulme A. C. (1968). Metabolic changes in excised fruit tissue-I. Factors affecting the development of a malate decarboxylation system during the ageing of disks of pre-climacteric apples. *Phytochemistry* 7 1439–1451. 10.1016/S0031-9422(00)88589-1

[B69] Ribeiro D. G. Cunha G. C. R. Santos C. Silva L. P. Oliveira Neto O. B. Labuto L. B. D. (2018). *Brassica oleracea* resistance-related proteins identified at an early stage of black rot disease. *Physiol. Mol. Plant Pathol.* 104 9–14. 10.1016/j.pmpp.2018.06.002

[B70] Rojas C. M. Senthil-Kumar M. Tzin V. Mysore K. S. (2014). Regulation of primary plant metabolism during plant-pathogen interactions and its contribution to plant defense. *Front. Plant Sci.* 5:17. 10.3389/fpls.2014.00017 24575102 PMC3919437

[B71] Rolland N. Curien G. Finazzi G. Kuntz M. Maréchal E. Matringe M. (2012). The biosynthetic capacities of the plastids and integration between cytoplasmic and chloroplast processes. *Annu. Rev. Genet.* 46 233–264. 10.1146/annurev-genet-110410-132544 22934643

[B72] Romano E. Vianna G. R. (2015). *Manual de Transformação Genética de Plantas.* Brasília: Embrapa-SPI.

[B73] Saha P. Kalia P. Sharma M. Singh D. (2016). New source of black rot disease resistance in *Brassica oleracea* and genetic analysis of resistance. *Euphytica* 207 35–48. 10.1007/s10681-015-1524-y

[B74] Santos C. Maximiano M. R. Ribeiro D. G. Oliveira-Neto O. B. Murad A. M. Franco O. L. (2017). Differential accumulation of *Xanthomonas campestris* pv. *campestris* proteins during the interaction with the host plant: contributions of an in vivo system. *Proteomics* 17:1700086. 10.1002/pmic.201700086 28471538

[B75] Sharma B. B. Kalia P. Yadava D. K. Singh D. Sharma T. R. (2016). Genetics and molecular mapping of black rot resistance locus Xca1bc on chromosome B-7 in ethiopian mustard (*Brassica carinata* A. Braun). *PLoS One* 11:e0152290. 10.1371/journal.pone.0152290 27023128 PMC4811439

[B76] Spallek T. Robatzek S. Göhre V. (2009). How microbes utilize host ubiquitination. *Cell. Microbiol.* 11 1425–1434. 10.1111/j.1462-5822.2009.01346.x 19523153

[B77] Stekhoven D. J. Omasits U. Quebatte M. Dehio C. Ahrens C. H. (2014). Proteome-wide identification of predominant subcellular protein localizations in a bacterial model organism. *J. Proteomics* 99 123–137. 10.1016/j.jprot.2014.01.015 24486812

[B78] Stintzi A. Heitz T. Prasad V. Wiedemann-Merdinoglu S. Kauffmann S. Geoffroy P. (1993). Plant ‘pathogenesis-related’ proteins and their role in defense against pathogens. *Biochimie* 75 687–706. 10.1016/0300-9084(93)90100-7 8286442

[B79] Swartzberg D. Kirshner B. Rav-David D. Elad Y. Granot D. (2008). *Botrytis cinerea* induces senescence and is inhibited by autoregulated expression of the IPT gene. *Eur. J. Plant Pathol.* 120 289–297. 10.1007/s10658-007-9217-6

[B80] Szalonek M. Sierpien B. Rymaszewski W. Gieczewska K. Garstka M. Lichocka M. (2015). Potato annexin STANN1 promotes drought tolerance and mitigates light stress in transgenic *Solanum tuberosum* L. plants. *PLoS One* 10:e0132683. 10.1371/journal.pone.0132683 26172952 PMC4501783

[B81] Taiz L. Zeiger E. Møller I. M. Murphy A. (2017). *Fisiologia e Desenvolvimento Vegetal.* Porto Alegre: Artmed Editora.

[B82] Takayama S. Sato T. Krajewski S. Kochel K. Irie S. Milian J. A. (1995). Cloning and functional analysis of BAG-1: a novel Bcl-2-binding protein with anti-cell death activity. *Cell* 80 279–284. 10.1016/0092-8674(95)90410-7 7834747

[B83] Tian S. Wang X. Li P. Wang H. Ji H. Xie J. (2016). Plant aquaporin AtPIP1; 4 links apoplastic H2O2 induction to disease immunity pathways. *Plant Physiol.* 171 1635–1650. 10.1104/pp.15.01237 26945050 PMC4936539

[B84] Ting J. T. L. Wu S. S. H. Ratnayake C. Huang A. H. C. (1998). Constituents of the tapetosomes and elaioplasts in *Brassica campestris* tapetum and their degradation and retention during microsporogenesis. *Plant J.* 16 541–551. 10.1046/j.1365-313x.1998.00325.x 10036772

[B85] Uberegui E. Hall M. Lorenzo Ó. Schröder W. P. Balsera M. (2015). An *Arabidopsis* soluble chloroplast proteomic analysis reveals the participation of the Executer pathway in response to increased light conditions. *J. Exp. Bot.* 66 2067–2077. 10.1093/jxb/erv018 25740923 PMC4378640

[B86] Untergasser A. Nijveen H. Rao X. Bisseling T. Geurts R. Leunissen J. A. M. (2007). Primer3Plus, an enhanced web interface to Primer3. *Nucleic Acids Res.* 35 W71–W74. 10.1093/nar/gkm306 17485472 PMC1933133

[B87] Valledor L. Weckwerth W. (2014). An improved detergent-compatible gel-fractionation LC-LTQ-Orbitrap-MS workflow for plant and microbial proteomics. *Methods Mol. Biol.* 1072 347–358. 10.1007/978-1-62703-631-3_25 24136534

[B88] Verkman A. S. (2013). Aquaporins. *Curr. Biol.* 23 R52–R55. 10.1016/j.cub.2012.11.025 23347934 PMC3590904

[B89] Villeth G. R. C. Carmo L. S. T. Silva L. P. Santos M. F. De Oliveira Neto O. B. Grossi-De-Sá M. F. (2016). Identification of proteins in susceptible and resistant *Brassica oleracea* responsive to *Xanthomonas campestris* pv. *campestris* infection. *J. Proteomics* 143 278–285. 10.1016/j.jprot.2016.01.014 26825537

[B90] Vogel C. Marcotte E. M. (2012). Insights into the regulation of protein abundance from proteomic and transcriptomic analyses. *Nat. Rev. Genet.* 13 227–232. 10.1038/nrg3185 22411467 PMC3654667

[B91] Walter M. H. Grima-Pettenati J. Grand C. Boudet A. M. Lamb C. J. (1988). Cinnamyl-alcohol dehydrogenase, a molecular marker specific for lignin synthesis: cDNA cloning and mRNA induction by fungal elicitor. *Proc. Natl. Acad. Sci.* 85 5546–5550. 10.1073/pnas.85.15.5546 3041415 PMC281795

[B92] Wang H. Hanash S. (2003). Multi-dimensional liquid phase based separations in proteomics. *J. Chromatogr. B* 787 11–18. 10.1016/S1570-0232(02)00335-5 12659729

[B93] Wang X. Komatsu S. (2016). Plant subcellular proteomics: application for exploring optimal cell function in soybean. *J. Proteomics* 143 45–56. 10.1016/j.jprot.2016.01.011 26808589

[B94] Weissbach L. Settleman J. Kalady M. F. Snijders A. J. Murthy A. E. Yan Y.-X. (1994). Identification of a human rasGAP-related protein containing calmodulin-binding motifs. *J. Biol. Chem.* 269 20517–20521. 8051149

[B95] Yamada K. Saijo Y. Nakagami H. Takano Y. (2016). Regulation of sugar transporter activity for antibacterial defense in *Arabidopsis*. *Science* 354 1427–1430. 10.1126/science.aah5692 27884939

[B96] Yamaguchi Y. Pearce G. Ryan C. A. (2006). The cell surface leucine-rich repeat receptor for AtPep1, an endogenous peptide elicitor in *Arabidopsis*, is functional in transgenic tobacco cells. *Proc. Natl. Acad. Sci. U.S.A.* 103 10104–10109. 10.1073/pnas.0603729103 16785433 PMC1502513

[B97] Zhang J. Xin L. Shan B. Chen W. Xie M. Yuen D. (2012). PEAKS DB: de novo sequencing assisted database search for sensitive and accurate peptide identification. *Mol. Cell. Proteomics* 11:M111.010587. 22186715 10.1074/mcp.M111.010587 PMC3322562

[B98] Zhang J. Zhou J.-M. (2010). Plant immunity triggered by microbial molecular signatures. *Mol. Plant* 3 783–793. 10.1093/mp/ssq035 20713980

[B99] Zhu W. Magbanua M. M. White F. F. (2000). Identification of two novel hrp-associated genes in the hrp gene cluster of *Xanthomonas oryzae* pv. *oryzae*. *J. Bacteriol.* 182 1844–1853. 10.1128/JB.182.7.1844-1853.2000 10714988 PMC101866

