# Corrigendum: Proteomic Analysis and Functional Validation of a *Brassica oleracea* Endochitinase Involved in Resistance to *Xanthomonas campestris*

**DOI:** 10.3389/fpls.2020.00201

**Published:** 2020-02-26

**Authors:** Cristiane Santos, Fábio C. S. Nogueira, Gilberto B. Domont, Wagner Fontes, Guilherme S. Prado, Peyman Habibi, Vanessa O. Santos, Osmundo B. Oliveira-Neto, Maria Fatima Grossi-de-Sá, Jesus V. Jorrín-Novo, Octavio L. Franco, Angela Mehta

**Affiliations:** ^1^Embrapa Recursos Genéticos e Biotecnologia, Brasília, Brazil; ^2^Departamento de Biologia, Universidade Federal de Juiz de Fora, Juiz de Fora, Brazil; ^3^Proteomics Unit, Chemistry Institute, Universidade Federal do Rio de Janeiro, Rio de Janeiro, Brazil; ^4^Departamento de Biologia Celular, Universidade de Brasília, Brasília, Brazil; ^5^Department of Bioprocess Engineering and Biotechnology, Universidade Federal do Paraná, Curitiba, Brazil; ^6^Departamento de Bioquímica e Biologia Molecular, Escola de Medicina, Faculdades Integradas da União Educacional do Planalto Central, Brasília, Brazil; ^7^Centro de Analises Proteomicas e Bioquimica, Pós-Graduação em Ciências Genômicas e Biotecnologia, Universidade Católica de Brasília, Brasília, Brazil; ^8^Department of Biochemistry and Molecular Biology, Universidad de Córdoba, Córdoba, Spain; ^9^S-Inova Biotech, Pós-Graduação em Biotecnologia, Universidade Católica Dom Bosco, Campo Grande, Brazil

**Keywords:** LC-MS/MS, differential protein abundance, qRT-PCR, gene overexpression, plant–pathogen interaction

In this original article, there was a mistake in Figure 4, and its legend, as published. One of the plant images is duplicated. The corrected Figure 4 is attached below. The correct caption also appears below, where a revision was made to change *BoCHIB4* for *BoCHB4*.

**Figure 4:**
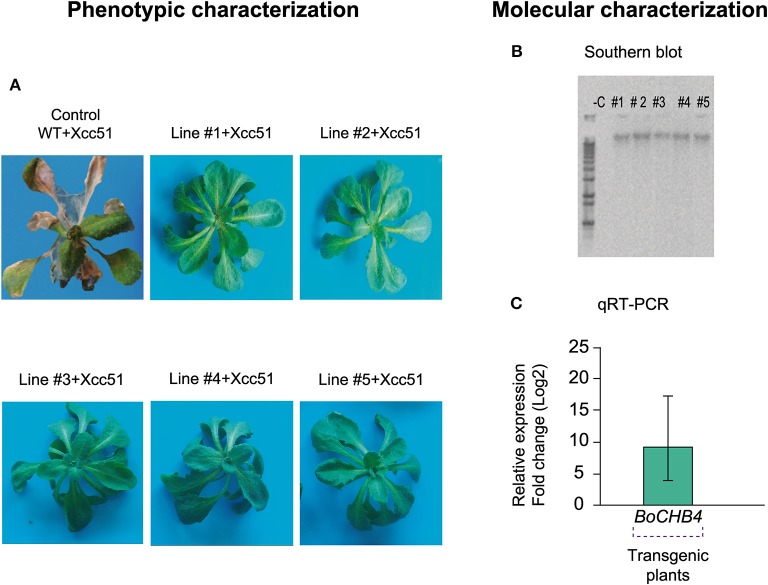
Phenotypic and molecular evaluation of transgenic lines and wild type (WT) *Arabidopsis thaliana* plants. **(A)** *A. thaliana* plants (WT and #1–5 *BoCHB4* lines) inoculated with *Xanthomonas campestris* pv. *campestris* (Xcc 51) at 5 days after inoculation (dai). **(B)** Southern blot analysis of *A. thaliana* transgenic lines (# 1–5) with *BoCHB4* radiolabeled probe. **(C)** Relative expression of *BoCHB4* gene in *A. thaliana* WT and transgenic lines (# 1–5).

The correct Figure 4 caption is:

Phenotypic and molecular evaluation of transgenic lines and wild type (WT) *Arabidopsis thaliana* plants. **(A)** *A. thaliana* plants (WT and #1–5 *BoCHB4* lines) inoculated with *Xanthomonas campestris* pv. *campestris* (Xcc 51) at 5 days after inoculation (dai). **(B)** Southern blot analysis of *A. thaliana* transgenic lines (# 1–5) with *BoCHB4* radiolabeled probe. **(C)** Relative expression of *BoCHB4* gene in *A. thaliana* WT and transgenic lines (# 1–5).

The authors apologize for this error and state that this does not change the scientific conclusions of the article in any way. The original article has been updated.

