# Supplementary figures and images for: Proteomic Analysis and Functional Validation of a Brassica oleracea Endochitinase Involved in Resistance to Xanthomonas campestris
Source: Front Plant Sci. 2019 Apr 12;10:414. doi: 10.3389/fpls.2019.00414 (PMC6473119; doi:10.3389/fpls.2019.00414)

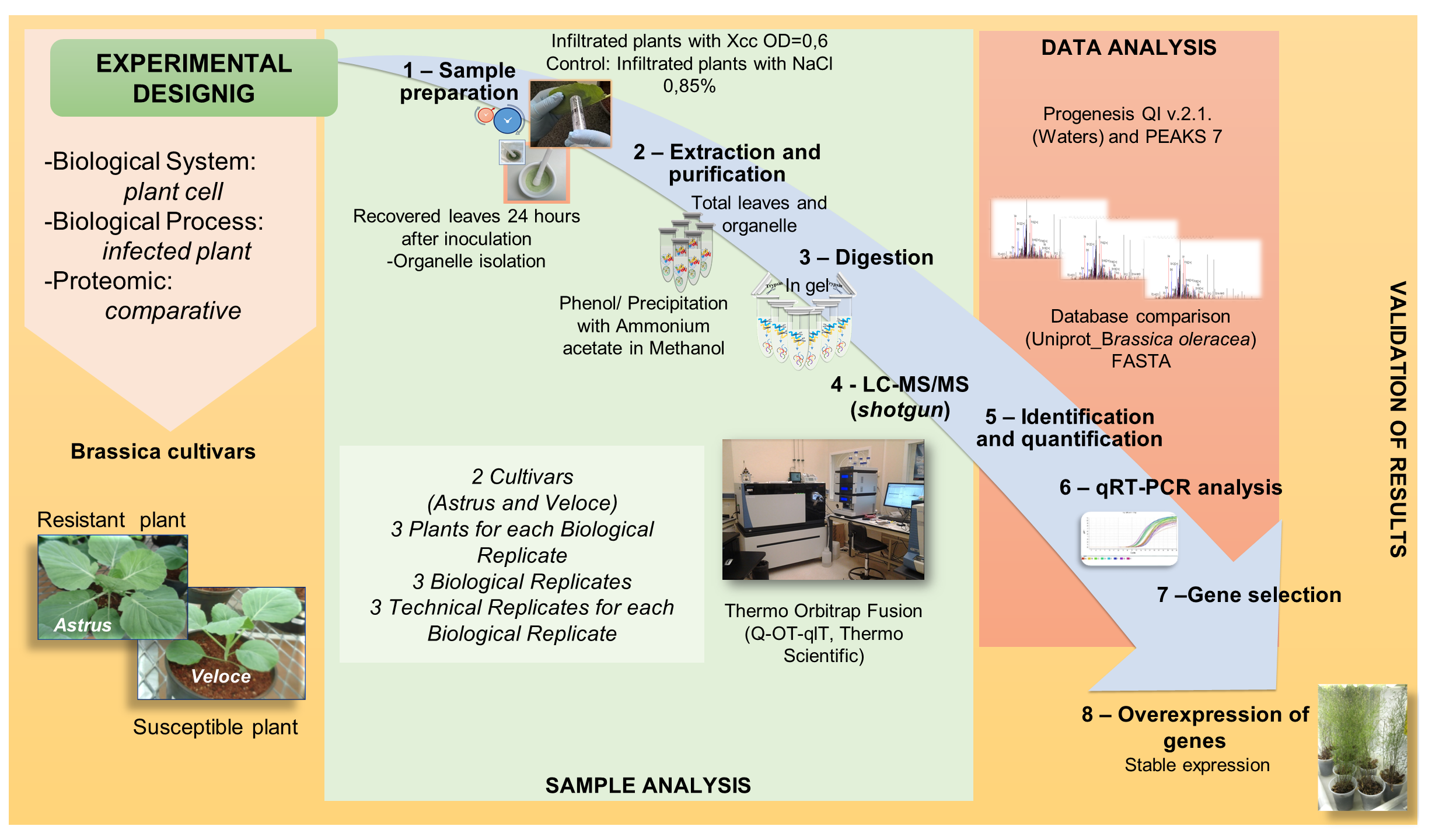

Supplement: FIGURE S1 — Workflow showing the entire procedure for sample preparation. [file Image_1.TIF]

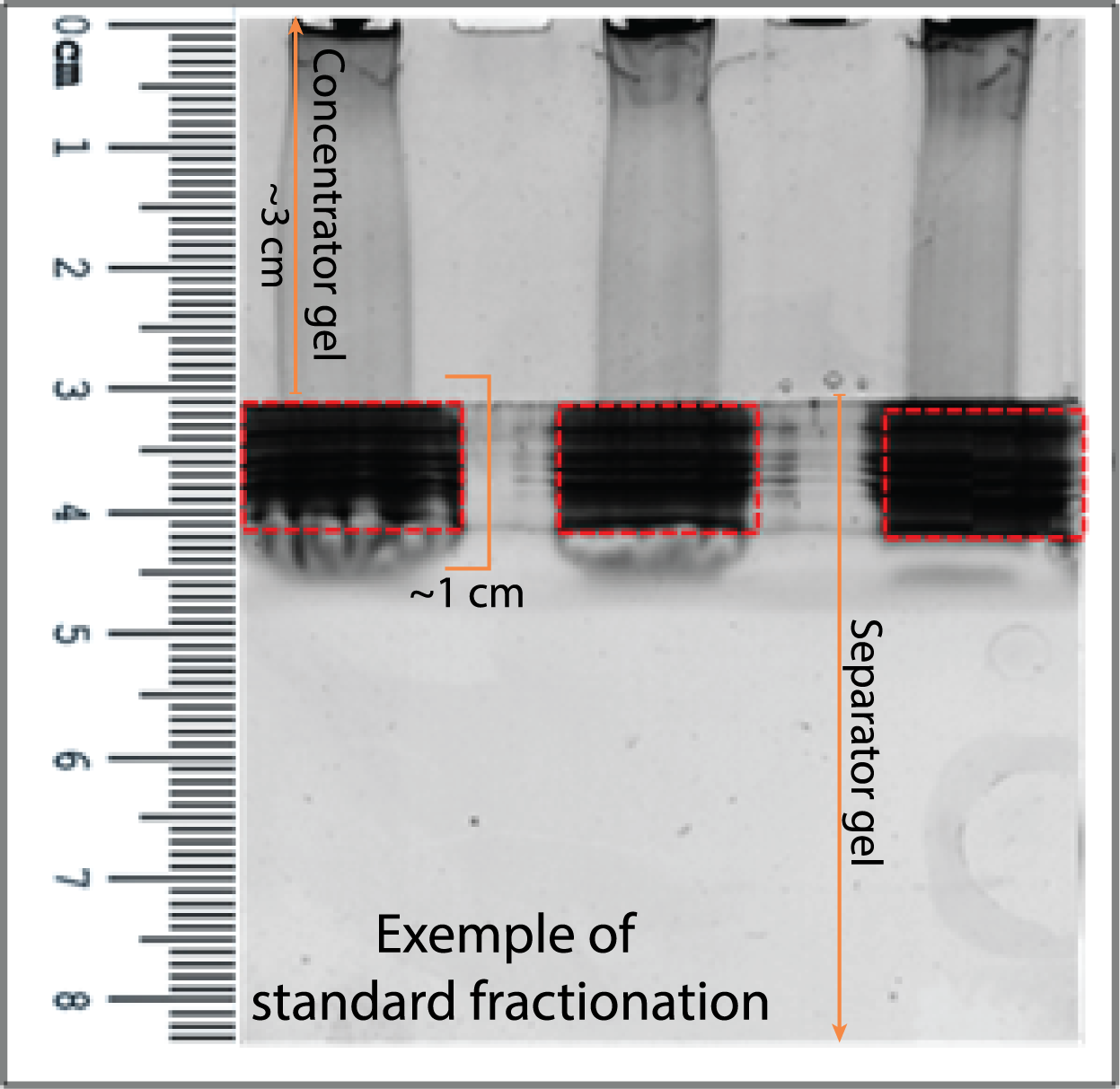

Supplement: FIGURE S2 — SDS-PAGE step prior to LC-MS/MS analysis. The section indicated by the red square was excised and submitted to in gel digestion using trypsin. [file Image_2.TIF]

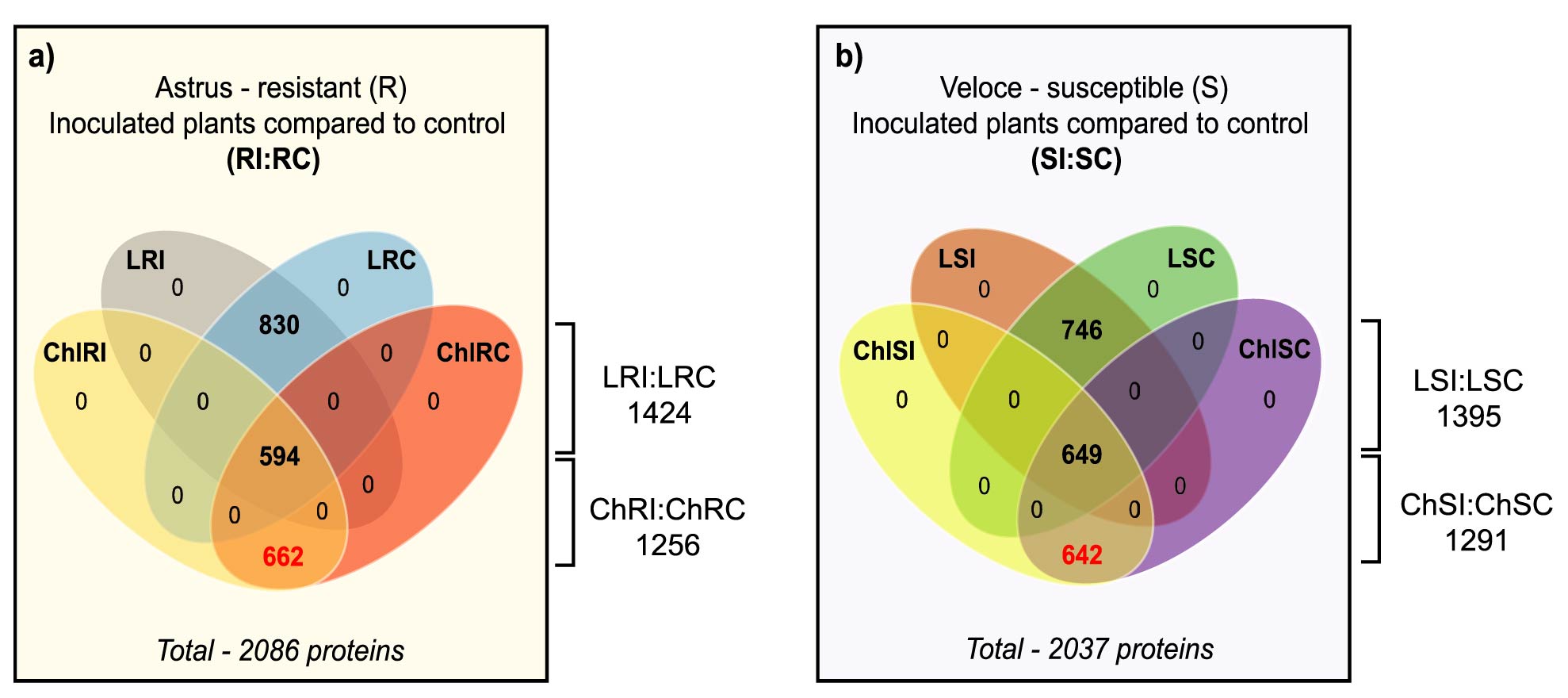

Supplement: FIGURE S3 — Venn diagram showing the total number of proteins identified by the proteomic analysis. (a) Quantitative distribution of proteins from inoculated leaves (LRI) and from the control condition (LRC) as well as from chloroplast-enriched inoculated samples (ChlRI) and from the control condition (ChlRC) in the resistant interaction. (b) Quantitative distribution of proteins from the susceptible inoculated leaves (LSI) and from the control condition (LSC) as well as from chloroplast-enriched inoculated samples (ChlSI) and from the control condition (ChlSC) in the susceptible interaction. [file Image_3.TIF]

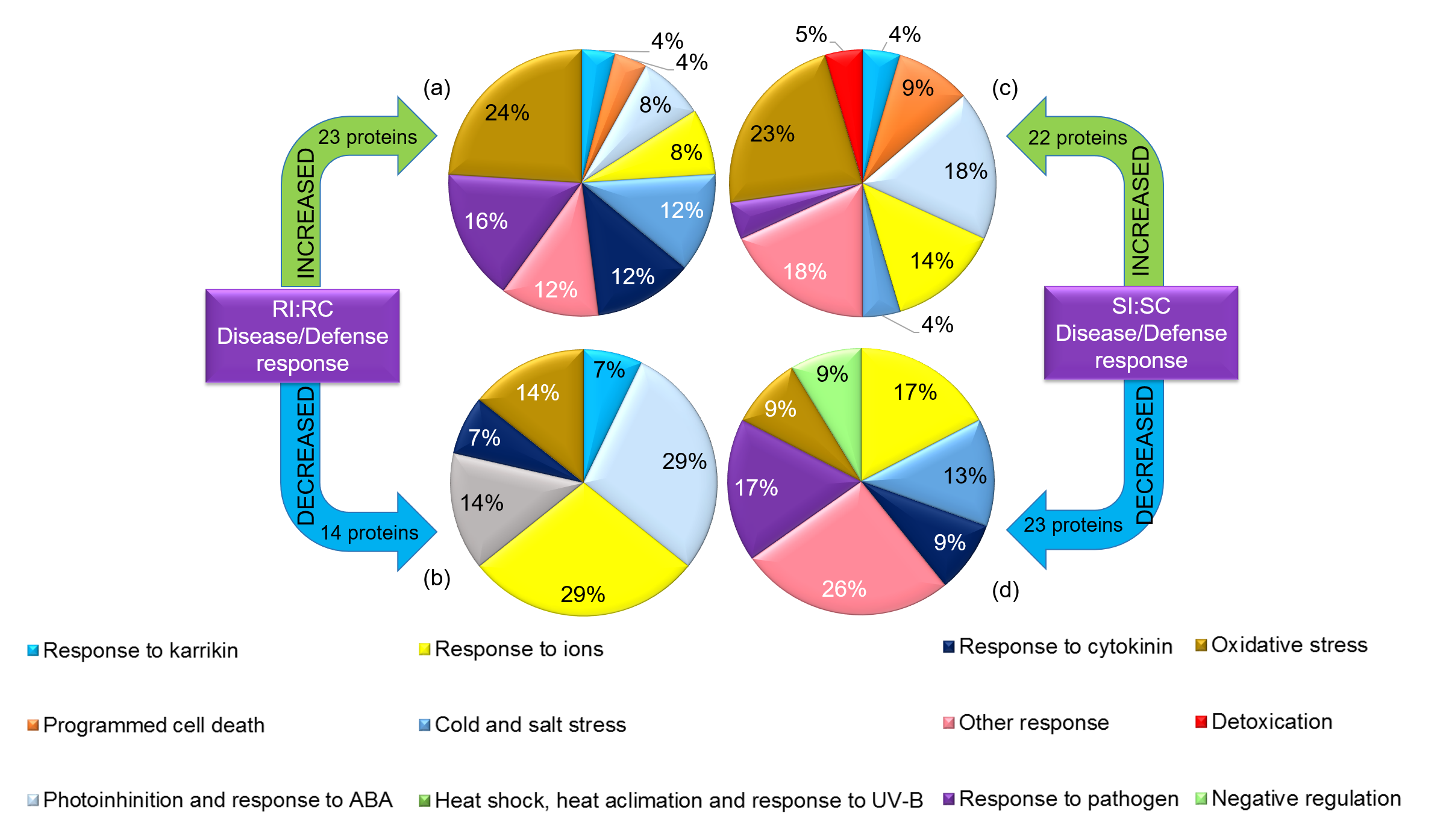

Supplement: FIGURE S4 — Subclassification of proteins related to disease/defense response, differentially abundant in resistant and susceptible cultivars. (a) Proteins with increased and (b) decreased abundance in the RI:RC comparison. (c) Proteins with increased and (d) decreased abundance in the SI:SC comparison. RI, resistant cultivar inoculated; RC, resistant cultivar control; SI, susceptible cultivar inoculated; SC, susceptible cultivar control. [file Image_4.TIF]
