# Supplementary material for: Proteomic Analysis and Functional Validation of a Brassica oleracea Endochitinase Involved in Resistance to Xanthomonas campestris
Source: Front Plant Sci. 2019 Apr 12;10:414. doi: 10.3389/fpls.2019.00414 (PMC6473119; doi:10.3389/fpls.2019.00414)
Supplement: TABLE S1 — Primers designed for the genes encoding the identified proteins used in qRT-PCR. [file Table_1.DOCX]

**Supplementary Table S1.** Primers designed for the genes encoding the identified proteins used in qRT-PCR.

| GeneBank^a^ | Protein Name | Primer Information (qRT-PCR) | | | |
| --- | --- | --- | --- | --- | --- |
|  |  | **Forward (5’-3’)**  **Reverse (5’-3’)** | **TM**  **(ºC)** | **pb^b^** | **Efficiency** |
| GAQY01006468.1 | Ankyrin repeat domain-containing protein 2-like *(BoAKR2A*) | CGTGGAGATGTTGAGGGTTT GCCGTATCCACAAGCAAAAT | 60.0  60.0 | 105 | 0.8403 |
| GAQY01000191.1 | Annexin (*BoANN2*) | AAATGGGAACTGACGAGTGG TCCTGCGCTGATACTCCTCT | 60.0  60.1 | 93 | 0.8553 |
| GAQY01037035.1 | Apyrase 5 (Bo*APY5*) | CTTGGGTGGTTGCGAACTAT CGTACGAGAGAACCCAGGAG | 60.0  59.9 | 140 | 0.8441 |
| GAQY01000707.1 | Aquaporin PIP1b1 (*BoPIP1-1*) | CCCTATTGGATTCGCTGTGT AGAGTTTTGGAACCGTGGTG | 60.0  60.0 | 96 | 0.8631 |
| GAQY01003687.1 | ATP-dependent Clp protease proteolytic subunit (*BoCLPP*) | CGAGCAATGGGCTGTTTTAT TGAAGCTGCTTTGCTTCTGA | 60.1  60.0 | 90 | 0.8912 |
| GAQY01035111.1 | ATP synthase gamma chain 1, chloroplastic (*BoATPC1*) | TTCCTCTCCCAAACCACAAC GGCTTCGGTGATCTTCTGAG | 59.9  60.0 | 125 | 0.8925 |
| GAQY01036959.1 | BAG family molecular chaperone regulator 7-like (Bo*BAG7*) | GAGTGGGGAAGGTGATGAGA CACCGAGAAACCTCCATTGT | 60.0  60.0 | 108 | 0.8589 |
| GAQY01039586.1 | Basic Endochitinase CHB4-like (*BoCHB4*) | CCTGATCCTAACCGTTTCCA CTTGCAAGGTCCTTCTTTGC | 59.9  60.0 | 136 | 0.8496 |
| AKM97935.1 | Cytochrome b559 subunit alpha (*BoPSBE*) | TGTCTGGAAGCACAGGAGAA AACCGGTGCTGACGAATAAC | 59.5  60.0 | 123 | 0.8505 |
| GAQY01005237.1 | Epithiospecifier -like (*BoESP*) | AGACAAACCTTCTGCGAGGA TCCAAAGCAAACCCTTCATC | 60.0  60.1 | 138 | 0.8491 |
| GAQY01006041.1 | Eukaryotic peptide chain release factor subunit 1-3 (*BoERF1-3*) | CTCTGCGACAACAAGTTCCA TCCCACTCAAAGTCCCAAAC | 60.0  59.9 | 115 | 0.8721 |
| GAQY01003838.1 | Gibberellin-regulated protein 1 (*BoGASA1*) | CCTAACCTTTGCCACAGAGC TAGCGTAGCACTGGCACTTG | 59.9  60.2 | 100 | 0.8369 |
| GAQY01002059.1 | Lectin-like protein At3g16530 (*BoLLP*) | CTTGTTCTTCCTCGGAGACG AATGGGATGGGGTTGATGTA | 60.0  59.9 | 132 | 0.8577 |
| GAQY01050797.1 | LRR receptor-like serine/threonine-protein kinase At1g29720 (*BoRFK1*) | GTCATGGAGCCTAGCCAATC TTCCTCCACCACGGATCTAC | 59.7  59.9 | 120 | 0.8731 |
| GAQY01001479.1 | Metacaspase-4 (*BoAMC4*) | TCATCATCCTCCCCTGTCTC CAAACCTACGGGGAAGAACA | 60.0  60.0 | 132 | 0.8332 |
| GAQY01017042.1 | MLP-like protein 31 (*BoMLP31*) | GAGCAACCTCGTCGCTAATC AAGCTTCTTGCTCACGATCC | 60.1  60.0 | 90 | 0.8417 |
| GAQY01042838.1 | Peroxiredoxin IIF, mitochondrial (*BoPRX2F*) | TGCCCAACCGTTTATGGTAT GCATGTGCCAAGCTACAAGA | 60.1  60.0 | 106 | 0.83 |
| GAQY01000503.1 | Peroxidase 32 (*BoPER32*) | TTGGTTCGTAAAAGGGTTGC CCTCCACTGCTGCTTTCATT | 60.0  60.4 | 138 | 0.8763 |
| GAQY01037531.1 | Photosystem II D2 protein (*BoPSBD*) | ACTTCCCCACCTAGCCACTT AGCCCAAAACTGCAGAAGAA | 60.0  60.0 | 116 | 0.7992 |
| GAQY01000204.1 | Protease Do-like 8, chloroplastic (*BoDEGP8*) | AGACGGCAAGATCCTTAGCA ACCCAATCCCTGGTGATGTA | 60.0  59.9 | 114 | 0.8518 |
| GAQY01019483.1 | Protein DETOXIFICATION (*BoDTX*) | AGTTTCGCTGCCCTTGACTA AACGGAGATACCGAGTGTGG | 60.0  60.0 | 141 | 0.8537 |
| GAQY01045530.1 | psbP domain-containing protein 4, chloroplastic (*BoPPD4*) | ATCAAACGCCATTTCTCAGG TGTGGATGAGGATGACAGGA | 59.9  60.0 | 106 | 0.882 |
| GAQY01034654.1 | Superoxide dismutase (*BoFSD1*) | AAATGGCTGCTTCAAGTGCT CATAAACGGCTCCAAAGCAT | 60.0  60.1 | 83 | 0.8507 |
| GAQY01042895.1 | Thylakoid lumenal 17.4 kDa protein, chloroplastic (*BoTL17*) | CTTCGCTTCCTGTCCAGTTC AAGATCTCTGCTCCGTGGAA | 60.0  60.0 | 90 | 0.8355 |
| GAQY01000155.1 | Translation initiation factor IF-2 (*BoIF-2*) | AACTGTCATGCTCGTTGCTG TCTTCTCAACGTGCCCTTCT | 60.1  60.0 | 142 | 0.8833 |
| GAQY01002318.1 | UDP-arabinopyranose mutase 1-like (*BoRGP1*) | CTGGCTCAACATCCCTGATT AGGCCAAGTTCATTCCACAC | 60.1  60.0 | 134 | 0.849 |
| GAQY01002282.1 | Uncharacterized protein At5g02240 (*At5g02240*) | GATTTGTTCCACCCATGGAC GATTGGATTGGCCAGAAGAA | 60.0  60.0 | 91 | 0.8558 |
| GAQY01044825.1 | Uncharacterized protein Ycf54 (*BoYCF54*) | TCCCCAAGATCACTCAAAGG GTCGCTTCAAAGGAATCAGC | 60.0  60.0 | 121 | 0.8564 |
| GAQY01004057.1 | Rubredoxin_like, 1 (*BoENH1*) | CCTCCTTCCACACCAGTGAT ACAGCAGCTTCGAAGACCAT | 60.0  60.0 | 104 | 0.8444 |
| GAQY01006072.1 | Universal stress protein YxiE-like (*BoUSPA*) | TGACATGCTCGACACTGGTT CCTCTGCTTCCCATGACAAT | 60.3  60.1 | 135 | 0.8753 |

Continuation

| GeneBank*^a^* | Protein name | Primers Informations | | | |
| --- | --- | --- | --- | --- | --- |
|  |  | **Forward (5’-3’)**  **Reverse (5’-3’)** | **TM**  **(ºC)** | **pb^b^** | **Efficiency** |
| AT3G18780 | ^£1^Actin *(ACT2*)* | CTTGCACCAAGCAGCATGAA  CCGATCCAGACACTGTACTTCCTT | 60.0  60.0 | 68 | 0.857 |
| AT5G60390 | ^£^Elongation factor 1- α (*EF-1α*)* | TGAGCACGCTCTTCTTGCTTTCA GGTGGTGGCATCCATCTTGTTACA | 60.0  60.0 | 76 | 0.859 |
| XM_013776124.1 | ^£^SAND family protein (*SAND*) | GGTAAAATCGGTGTGGCTTC | 60.0 | 171 | 0.842 |
|  |  | ATGGCGACTTCAGATTCGAG | 60.0 |  |  |
| XM_013728490.1 | ^£^*TATA-box-binding protein 1* (*TBP1*) | TCTTGGCTCCAGTACAAACC | 60.0 | 177 | 0.8584 |
|  |  | ACATTGTGTCGACGGTGAAC | 60.0 |  |  |
| XM_013755988.1 | ^£^Tubulin beta-6 (*TUB6*) | TCCATCTCGTCCATTCCTTC | 60.0 | 179 | 0.860 |
|  |  | AACGTGAAGTCAAGCGTGTG | 60.0 |  |  |
| XM_013778573.1 | ^£^Ubiquitin-60S ribosomal protein L40 Ubiquitin (*UBQ1*) | GGCCTTGTATAATCCCTGATGAATAAG AAAGAGATAACAGGAACGGAAACATAGT | 60.0  60.0 | 186 | 0.8354 |

^a^Identification number available in NCBI database; ^b^Amplicon size (base pairs); *Brassica oleracea* (*Bo*) gene name based on the homologue in *Arabidopsis thaliana;* ^£^Reference genes from *B. oleracea* or *A. thaliana* (*).
